# The “Dark Side” of Community Ties: Collective Action and Lynching in Mexico

**DOI:** 10.1177/00031224241253268

**Published:** 2024-06-07

**Authors:** Enzo Nussio

**Affiliations:** a ETH Zurich

**Keywords:** lynching, Mexico, community, social ties, social capital, trust, collective action, violence

## Abstract

Lynching remains a common form of collective punishment for alleged wrongdoers in Latin America, Africa, and Asia today. Unlike other kinds of collective violence, lynching is usually not carried out by standing organizations. How do lynch mobs overcome the high barriers to violent collective action? I argue that they draw on local community ties to compensate for a lack of centralized organization. Lynch mobs benefit from solidarity and peer pressure, which facilitate collective action. The study focuses on Mexico, where lynching is prevalent and often amounts to the collective beating of thieves. Based on original survey data from Mexico City and a novel lynching event dataset covering the whole of Mexico, I find that individuals with more ties in their communities participate more often in lynching, and municipalities with more highly integrated communities have higher lynching rates. As community ties and lynching may be endogenously related, I also examine the posited mechanisms and the causal direction. Findings reveal that municipalities exposed to a recent major earthquake—an event that tends to increase community ties—subsequently experienced increased levels of lynching. Importantly, I find that interpersonal trust is unrelated to lynching, thus showing that different aspects of social capital have diverging consequences for collective violence, with community ties revealing a “dark side.”

More than 100 countries around the globe have registered instances of lynching in recent times (Jung and Cohen 2020). Guatemala, South Africa, and Indonesia, for example, often see mobs taking justice into their own hands, challenging the foundations of state authority and effectively replacing its functions (Smith 2019). Lynch mobs are rarely identified as political agents, perhaps because they do not systematically attack state representatives nor follow clear ideological precepts. However, contemporary lynchings represent a political expression of the marginalized, an “avenue for the communication of grievances” and the restoration of justice (Goldstein 2003), akin to Thompson’s (1971) food riots and Scott’s (1976) peasant rebellions. Lynching can thus be classified as a form of political violence (Kalyvas 2019).

Unlike more commonly studied actors of political violence like insurgents, terrorists, and militias, lynch mobs do not usually count on standing organizational structures to facilitate collective action. Collective action for the purpose of a collective good, like security or justice, is hard. Classical social theory holds that if everybody can reap the same benefits as those who participate, there is no incentive to personally engage in the creation of a collective good (Olson 1965).^1^ This collective action problem is more severe when the personal costs of participation are particularly high, as in the case of violent collective action (Wood 2003). Armed organizations typically overcome this high barrier for violent collective action by use of material incentives (exclusively available to their members), coercion (raising the costs for non-participation), and appeals to ideology and identity (creating nonmaterial incentives) (Lichbach 1995; Nussio and Ugarriza 2021). These tools of centralized and standing organizations are not usually available to lynch mobs. Hence, how can they overcome the barriers to violent collective action?

Previous research on lynching has rightly focused on state weakness and illegitimacy as enabling factors (Jung and Cohen 2020; Kloppe-Santamaría 2020; Pfeifer 2004; Smith 2019). Other research has centered on violations to moral beliefs and outrage (Asif and Weenink 2022; Nussio 2023; Nussio and Clayton 2023), the meaning-making functions of lynching rituals (Ball 1994; Fujii 2021), and the legacies of past violence (Bateson 2013; Rickard and Bakke 2021). Most literature on lynching focuses on the historical U.S. case and is dominated by race-based explanations related to the status of whites (Epperly et al. 2020; Smångs 2017; Tolnay and Beck 1995; Wells 1892), as well as cultural explanations related to Southern honor (Brundage 1993; Wyatt-Brown 1982). Researchers focusing on Indonesia have emphasized the importance of political and economic crisis as accelerators of lynching violence (Colombijn 2002; Tajima 2014; Welsh 2008). These explanations account for variation in lynching across contexts and periods, but they do not address the collective action problem.

In this article, I argue that, to substitute the functions of centralized organization, lynching draws on social ties within local communities, meaning the manifold and often intimate connections in a tightly-knit web of neighborhoods and villages (Taylor 1982). Lynchings proceed “when people become convinced that enough others are participating” (Marwell and Oliver 1993:1), and community ties are what make this process possible. Ties between community members can be beneficial for cooperation, for example, when neighbors help each other out, know about each other’s worries, protest for public service provision, and prevent crime together (Ley 2022; Sampson 2008; Sharkey, Torrats-Espinosa, and Takyar 2017), but these ties also have a “dark side” (Cooney 1998), as they can facilitate illegal and violent collective action. Hence, lynch mobs do not draw on standing and centralized organizational structures to solve the collective action problem; rather, they use community ties to help in the “process of organizing” (Ostrom 1990:39).

I argue that community ties build the foundation for the interrelated social forces of solidarity and peer pressure, which facilitate the process of organizing and enable lynching. Solidarity is a feeling of unitedness. It creates nonmaterial incentives for cooperation and a shared expectation that closely tied neighbors have each other’s back. Peer pressure generates reputational costs for non-cooperativeness, as community ties fuel the circulation of gossip and rumors. This pressure pushes individuals to behave in line with local norms. The resulting mobilization capacity of a community with manifold ties produces asymmetry between community members and wrongdoers, conducive to the use of violence (Collins 2008). In the absence of manifold ties, locals are unable to overcome the collective action problem. Community ties are thus a key driver of lynching.

Community ties provide a resource for collective action, but they do not, in themselves, provide a motivation for the use of violence. I thus see this argument as complementary to existing theories on state illegitimacy, collective emotions, and morality, which are sources of motivation and provide additional benefits for participants. Most importantly, a weak state creates opportunity for non-state violence, motivates citizens to take justice into their own hands, and makes violent self-justice a legitimate practice (Cruz and Kloppe-Santamaría 2019; Jackson et al. 2013; Nivette 2016; Tankebe 2009; Zizumbo-Colunga 2017). In this context, lynching can become a common and accepted repertoire of social control (Colombijn 2002). Lynching is not the only method of social control; communities have many methods to regulate behavior, including nonviolent methods (Taylor 1982). In this study, I only observe lynching, perhaps the most extreme and rarely used method.

The presented argument is applicable to contexts where state capacity is limited, and where lynching is a widely known tool of social control. Within such contexts, I expect community ties to explain the variation in individual participation in lynching and the aggregate likelihood of lynching events. In contrast, I expect that another often studied aspect of social capital—trust placed in others (Putnam 1993)—does not produce the same effect, as lynching requires highly localized and mobilizable resources, which facilitate the process of organizing and go beyond evaluations of others’ benevolence (Luhmann 2000). Comparing the effects of community ties with the weak tie implied by generalized trust allows me to examine the specificity of my argument.

The geographic context for this study is Mexico, where lynching is a common and widely accepted practice (CNDH 2019; Kloppe-Santamaría 2020), in part due to a historical sense of impunity and weak administration of justice in several regions of the country (Piccato 2017). According to an original dataset created for this study (Nussio and Clayton 2024), Mexico is the country in Latin America with the largest absolute number of fatal and non-fatal lynchings (Guatemala and Bolivia have higher lynching rates). In this article, I understand lynching as publicly displayed physical violence against alleged wrongdoers perpetrated by a group of civilians. This definition is in line with scholarship on contemporary lynching in Latin America and other world regions, which focuses not on the outcome but on the act of lynching (Colombijn 2002; Godoy 2006; Kloppe-Santamaría 2020). As I will discuss further, this focus is a deliberate deviation from the definitions of lynching used in the U.S. context, which are outcome-focused and define lynching as a killing (Waldrep 2000).

Lynchings in Mexico are historically concentrated in the center and south of the country (Kloppe-Santamaría 2020). Lynchings in Latin America and Mexico are often in response to a broader crime threat (Godoy 2006), which turns individual delinquents into “everybody’s thief” (Gamallo 2020:195). This is different from the United States where threats to the status of whites fueled lynchings (Tolnay and Beck 1995), or India where Hindu extremists feel threatened by Muslims who eat cow-meat (Varshney and Staggs 2024). The modal type of contemporary lynching in Mexico amounts to the collective beating of male thieves, although punishment of other forms of wrongdoing (alleged child abuse, reckless driving, murder, and kidnapping) and other forms of violence (forced detention and burning) are also common. According to news reports, in 81 percent of lynching cases, the victims are caught by the community in the act of committing an alleged wrongdoing, and in 17 percent of cases, at least one attacked person died because of the violence inflicted. As in the U.S. context (Smångs 2016), mob size varies considerably. In Mexico, mobs with more than 100 people (45 percent) are most common, and 39 percent of mobs have between 20 and 99 participants. Active collaboration of state agents with lynch mobs is rare, but mob participants are rarely arrested, suggesting some level of tolerance.

Given the difficulty of identifying the effects of community ties on lynching, my empirical analysis is designed to balance the advantages and limitations of different approaches. In a first step, I use an original survey conducted in Mexico City designed for this study. To capture participation in lynching, I refer to a vignette about a lynching-style incident. To capture community ties, I ask respondents how many people they know by name in their neighborhood; this is a novel way of inquiring about community ties. Statistical analysis indicates that community ties are associated with participation in lynching. In contrast, interpersonal trust is not positively related to participation, suggesting the association is specific to community ties. These findings are robust to other specifications and the use of proxies, but inherent limitations remain. For instance, the findings potentially suffer from endogeneity between community ties and lynching participation, as lynching may strengthen community ties (Colombijn 2002), and collective punishment may generate solidarity among the punishing mob (Garland 1990:23).

I therefore conduct a series of additional analyses. First, I study suggestive indicators of solidarity and peer pressure. This analysis shows that the posited mechanisms are plausibly related to community ties. Second, I designed a similar study on the aggregate level of Mexican municipalities, using an original lynching dataset based on news reports. I find a relationship between community ties, but not trust, and lynching rates, which means the results are consistent on the aggregate level and for the whole of Mexico. Third, to allow for cautious causal inference, I use the natural experiment of the 2017 earthquake in the region of Mexico City; I find that this solidarity-inducing shock was followed by increased levels of lynching in exposed areas. Despite the limitations of each approach, together they show a consistent picture: community ties are related to lynching.

This study makes two key contributions. First, it contributes to the literature on effects of social ties. Early proponents of social capital theory have praised social ties for their benefits for society and the polity (Coleman 1988; Putnam 1993), and the related collective efficacy theory shows a crime prevention effect (Sampson, Raudenbush, and Earls 1997). However, critical voices have warned about the “dark side,” or negative social effects, of social ties (Cooney 1998; Levi 1996; Ostrom 2000; Portes and Landolt 1996; Putzel 1997). Prior research shows that social capital can contribute to diverse forms of anti-social behavior and violence (Alcorta et al. 2020; Cooney 1998; Krakowski 2021; McDoom 2014; Nussio and Oppenheim 2014; Satyanath, Voigtländer, and Voth 2017; Scacco 2012). The study of lynching provides particularly strong evidence of this effect. Unlike many other forms of violence, lynching is localized, making the social structure in the location of lynching highly informative. Rebel and terrorist violence, for instance, occurs in places unconnected to perpetrators’ origins and responds to strategic targeting. In contrast, the localized nature of lynching facilitates the empirical identification of a connection between community ties and violence. Furthermore, the distinction between community ties and trust shows how different aspects of social capital have divergent consequences for collective violence (Putnam 2002).

Second, this study contributes to the understanding of lynching by drawing on classical social theory and introducing new empirical evidence. Prior research has speculated about a link between community ties and lynching (Godoy 2006; Goldstein et al. 2007; Smith 2019; Zizumbo-Colunga 2015, 2019), but this is the first study using original and fine-grained lynching participation and event data to examine this hypothesis. Unlike a growing body of research focusing on attitudes toward lynching (e.g., Dow et al. 2024; Freire and Skarbek 2023; Nivette 2016; Wilke 2022), this study delves deeper by analyzing actual participation in lynching. Using support as a proxy for participation is limited, as it captures preferences but not decision-making (Agostini and van Zomeren 2021; Granovetter 1978). By focusing on participation in lynching and lynching events, this study provides direct behavioral evidence and sheds light on how social ties facilitate overcoming the high barriers to violent collective action.

## Theory

### The Concept of Lynching

Lynching is understood here as *publicly displayed physical violence executed by a group of civilians against alleged wrongdoers.* There are thus four definitional criteria. First, lynching implies an *act of physical violence*, distinguishing it from rhetorical uses of the term, as in, for example, “social media lynching” (Olabuenaga 2019). The act of physical violence can, but does not need to, lead to a fatal outcome. Readers should note that this contrasts with definitions used in the U.S. context, which usually require a fatal outcome (Waldrep 2000). In line with many authors studying lynching outside the U.S. context and particularly Latin America (Berg and Wendt 2011; Castillo Claudett 2000; Colombijn 2002; Godoy 2006; Kloppe-Santamaría 2020; Vilas 2001), I focus on the act of violence rather than its outcome, which may depend on fortuitous circumstances. Focusing on the outcome rather than the act excludes cases where the target escaped, was rescued, or simply survived the attack, although an act of lynching was under way. U.S. researchers have called such lynchings without fatality “threatened” or “averted” lynchings (Beck, Tolnay, and Bailey 2016; Hagen, Makovi, and Bearman 2013).^2^

Second, perpetrators must act against *an alleged wrongdoing*. In lynchings, targeted individuals are held responsible for what they allegedly did. This distinguishes lynching from rioting or hate crimes, which do not require a particular wrongdoing by an individual, but often target individuals for the sake of belonging to a particular group (Horowitz 2001:22). Senechal de la Roche (1997:61) calls this aspect of lynching “individual liability.” I use the term wrongdoing instead of other commonly used terms with a legal connotation, such as offense or crime, as transgressions that lead to lynchings are not always illegal but may violate local norms embodied by the “collective conscience” of a community (Durkheim 1893). Wrongdoing thus captures a variety of transgressions going beyond the legal domain. I include the term “alleged” because attackers are acting under the assumption that a wrongdoing occurred without establishing its veracity. Alleged wrongdoing is a definitional criterion for lynching, but it is certainly not the sole reason for the selection of lynching targets (Martins 1991). In the U.S. South, for example, African Americans and outsiders in their respective communities (i.e., “marginal men”) were most often lynched (Bailey and Tolnay 2015).

Third, the act must include some form of *public display*, sometimes enacted as a spectacle (Fujii 2021). This criterion differentiates lynching from clandestine forms of collective violence like social cleansing. Ritualized actions may be part of lynching (Ball 1994), but they are not a definitional aspect, as lynchings vary so much that they cannot be generally classified as rituals. Lynchings can vary in levels of publicness (Smångs 2017), but they occur without any intention of concealing.

Fourth and most important for this article, lynching is perpetrated by a *group of civilians*, rather than members of a standing and centralized organization (Senechal de la Roche 1997). This criterion differentiates lynching from violence used by gangs, rebels, and regular security forces. The term “mob” is often used in this context, denoting a temporary and fickle group with an ambiguous agenda and a fluid, non-hierarchical membership (Snow and Moss 2014).^3^ It originates in the Latin expression *mobile vulgus*, meaning an excitable crowd. In line with this definition, lynching is most commonly a spontaneous form of collective violence (Senechal de la Roche 1996). This is in contrast to the concept of vigilantism, which involves a higher level of organization (Moncada 2017, 2023) although it may happen in similar contexts as lynching (Martinez 2018; Obert and Mattiacci 2018). Vigilantism also includes the prevention and investigation of violence (Bateson 2021). Lynching is thus the more precise term, as it denotes the act of violence, rather than a more sustained social activity, as implied by vigilantism.^4^

### Barriers to Violent Collective Action

The nature of lynching is puzzling. According to classical social theory, collective action for the purpose of collective goods, like justice, deterrence, or security, faces important barriers. If it is successful, benefits are nonexcludable, meaning they cannot be withheld from non-participants, and individuals must therefore be convinced that participating is preferable to free-riding (Olson 1965).^5^ For collective action that involves violence, this dilemma is even more pronounced due to the risks for one’s own physical integrity (Wood 2003) and our evolved aversion against confrontational tension and violence (Collins 2008; Cushman et al. 2012).

Other forms of political violence, like rebellion or terrorism, usually draw on standing organizations that have already solved their collective action problem and strategically resort to selective incentives (Weinstein 2007), coercion (Nussio and Ugarriza 2021), and normative commitment based on common ideology (Costalli and Ruggeri 2015) and group identification (della Porta 1995; Littman and Paluck 2015). Lynch mobs, in contrast, are formed ad hoc and thus lack the centralized and standing organizational structure that typically facilitates violent collective action.

Riots are perhaps most similar to lynching in lacking centralized organization and are thus similarly puzzling (Scacco 2012). However, riots are sometimes instrumentally instigated by political leaders and can provide selective incentives, such as opportunities to loot, or nonmaterial incentives related to identity-based grievances (Bulutgil and Prasad 2023; Horowitz 2001; Thompson 1971; Wilkinson 2009). According to Tilly (2003:18), the term “riot” is exclusively used by those who want to delegitimize violent protest. One could thus argue that riots are often not planned to be violent but may turn violent in reaction to repression.

These explanations for riots are of little help for understanding lynching. Most acts of contemporary lynching do not provide access to material incentives, and elites are not usually involved. In the Latin American and other non-U.S. contexts, grievance-based explanations related to ethnic and racial identity are also less relevant, as lynching happens mostly among co-ethnics (Berg and Wendt 2011; Freire and Skarbek 2023; Zizumbo-Colunga 2015). Furthermore, lynching is rarely a reaction to repression, but rather attracts state intervention. The puzzling question is thus: How is lynching, as violent collective action, possible?

Prior work notes that some individuals may be involved for the “process benefits” (Elster 2007:399). People may take pleasure in engaging as they feel a sense of agency (Wood 2003) or are attracted by the entertainment of a public spectacle (Thurston 2011). Such personal inclinations are common across localities but disconnected from social structure and thus carry little explanatory weight for spatial variation in collective action. Explanations that connect social structure with individual behavior are more relevant for our question.

### Community Ties, Solidarity, and Peer Pressure

I argue that community ties can provide a structure that substitutes for the functions of standing organization and enables violent collective action. By community ties, I mean connections between individuals sharing the same small-scale geographic space. These connections are often related to family relationships, friendship, and repeated interaction in close proximity, including rural villages and urban neighborhoods (Sampson 2008). The number of ties varies from one community to another. For Taylor (1982), the direct and many-sided relations between members who practice reciprocity are what define a local community.^6^ If this kind of structure exists, he argues, communities can create social order without state intervention, including the punishment of wrongdoing. This argument is similar to the predictions of collective efficacy theory, which claims that social cohesion is a key resource for effective crime control in urban neighborhoods (Sampson 2008).^7^ Communities with an abundance of ties can engage in the “process of organizing” (Ostrom 1990:39) and create a high level of localized interdependence (Marwell and Oliver 1993) without the existence of centralized and standing organization. Plentiful social ties can empower communities for self-governance and crime prevention, but these same connections can also enable lynching, a form of violent collective action.

I argue that community ties facilitate collective action through the interrelated social forces of solidarity and peer pressure. Solidarity provides nonmaterial incentives for participation in collective action, and peer pressure generates costs for non-participation. Together, these social forces steer individual behavior into collective action. In the absence of abundant community ties, individuals lack this source of guidance and collective action is unlikely.

Solidarity creates nonmaterial incentives for participation in collective activities, including collective violence (Barolsky 2016; della Porta 1995; Mukhopadhyay and Howe 2023). I understand solidarity as a reciprocal feeling of unitedness that binds members of the same community to each other (Jasper 2011). These feelings are stronger in a community with abundant ties, because of repeated contact and high levels of interdependence. Lynch groups often draw on family members, neighbors, residents, and co-workers from the nearby community (Smith 2019; Vilas 2001), where feelings of solidarity provide an incentive for participation. A billboard in San Luis Potosí, Mexico, in a neighborhood known for lynchings, appeals to exactly these feelings: “If you rob one of us, you rob all of us” (El Universal 2019). More than a century ago, Mead (1918) warned that the consequences of such solidarity, in suppressing concerns for the individual, could be disastrous, alluding to a “dark side” of solidarity.

Solidarity further underpins a shared expectation of mutual protection and insulation from retaliation. Community members can credibly convey the impression to each other that no negative consequences are to be feared when engaging in violent collective action—both from the attacked persons and from state agents who may arrest attackers (Elster 2007:392). The seventeenth-century Spanish playwright Lope de Vega (1619) presented a compelling illustration of this dynamic. Based on historical occurrences, he wrote about inhabitants of the town *Fuenteovejuna* who stoned their abusive local commander to death. When interrogated, every villager responded: “Fuenteovejuna did it.” They were saying the whole town killed the commander, effectively allowing for the diffusion of responsibility of each individual participant (Bandura 2016). Where community members expect their neighbors to participate and to follow the norm of not snitching (Bicchieri 2002), communities can quickly create extreme asymmetry between perpetrators and wrongdoers (Vilas 2001). The resulting situation of “attacking the weak” (Collins 2008) helps overcome individuals’ aversion to violence and allows community members to express “unlimited sadism” without risking retribution from the alleged wrongdoers or fear of legal punishment (Brundage 1993:42). In the absence of manifold local ties, community members cannot be sufficiently sure about their neighbors’ behavior and thus abstain from participation.

In addition to solidarity, community ties are also the foundation of peer pressure. Peer pressure raises costs of non-participation and pushes people to fall in line with local norms (Petersen 2001; Scacco 2012). It instills a “sense of should” responsive to reputational costs (Theriault, Young, and Barrett 2021). Individuals adjust their behavior to demonstrate cooperativeness and to show intolerance of non-cooperativeness. With behavior favoring one’s community, members can cultivate a reputation for group favoritism (Voswinkel 2011). Such a reputation is then cemented with informal communication, including rumors (Herriman 2010) and gossip (Giardini and Wittek 2019).

In closely tied communities, rumors travel easily, facilitating violence (Cooney 1998; Smith 2019). Collins (2008:118) notes how rumors fuel peer pressure: “To credit the rumor is to show oneself a member of the group; . . . to reject it is to put yourself outside and in opposition to the group” (see also Kloppe-Santamaría 2021). Approving rumors thus demonstrates community members’ loyalty with the group. Mark Twain (1923) identified the related fear of a neighbor’s disapproval, “more dreaded than wounds and death,” as a source for lynchings in the southern United States. This process can be accelerated by the presence of local leaders, acting as “large contributors” to collective action (Marwell and Oliver 1993), who mobilize their neighbors and friends (Benevides and Fischer 1991; Welsh 2008). Such individuals are particularly responsive to peer pressure, as they are susceptible to protecting their popularity in the community (Haynie 2001:1026; Kovács, Hsu, and Sharkey 2023; Littman and Paluck 2015).

Not only can peer pressure drive mobilization into lynching, it also elevates the costs of raising dissent once a lynching is under way, as mobs punish accomplices of alleged wrongdoers (Handy 2004). For members of heavily tied communities, shunning, ostracism, withdrawal of reciprocal aid, and even violence may be the consequence of non-participation or interference with the group (Eriksson, Strimling, and Gelfand 2021; Taylor 1982). Without manifold community ties, local peer pressure to enforce social norms has less grip on individuals as reputational costs are less relevant. Hence, violent collective action is less likely in less integrated communities.

### Implications and Scope Conditions

**Figure 1.**
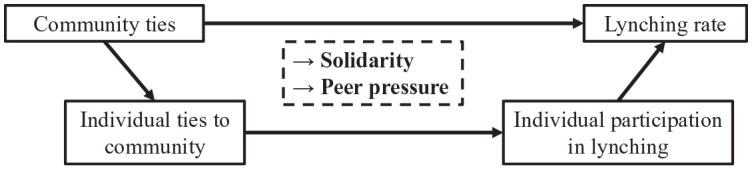
Boat Model of Community Ties and Lynching

Figure 1 summarizes the presented argument using Coleman’s (1990) boat model. The upper part of the boat shows the connection between community ties and lynching on the aggregate level. The lower part of the boat shows how an individual’s participation in lynching depends on their ties to the community. Individuals who have manifold connections to their communities are more likely to develop feelings of solidarity (a nonmaterial incentive for participation) and to be exposed to peer pressure (generating costs for non-participation).

In summary, I hypothesize that *community ties facilitate lynching*. This hypothesis refers both to aggregate units and individuals, as reflected in the two levels of the boat model. Individuals who have more ties to their local community should be more likely to participate in lynching, and aggregate units with more abundant community ties should see higher lynching rates.

Observing a difference between the aggregate and individual levels of observation would imply that my argument is incorrectly specified. For example, it is possible that individuals who have more ties to their community are more likely to participate in lynchings, but that on the aggregate level, there is no relationship between community ties and lynching rate. In this case, my argument would explain individual behavior but not aggregate-level variation. Also, if this study only focused on the aggregate level, I would not be able to make inferences about individual behavior, due to the risk of ecological fallacy (Robinson 1950). The combination of the two levels of analysis thus provides a stronger test of my argument.

In contrast to community ties, I expect that another aspect of social capital often discussed in the literature—interpersonal trust—should not produce the same effect. Trust is the expectation that others will be benevolent and not cause me harm, and it is based on prior experience (Luhmann 2000). Trust by this definition amounts to a weak tie beneficial for bridging across groups (Granovetter 1973) but insufficient to generate the localized process of organizing necessary to engage in violent collective action. Studying the consequences of both community ties and trust allows us to identify whether lynching is specifically related to community ties or relies on any form of social capital.

The proposed argument applies within the following scope conditions. First, the described social forces can only facilitate lynching in contexts where lynching is a known and accepted practice of social control (Colombijn 2002). Repertoires of violence are context-dependent, and dealing with wrongdoing may thus vary from one context to another (Gamallo 2015; Gutiérrez-Sanín and Wood 2017). In certain contexts, lynching is inconceivable even though the social conditions may be ripe. Only in contexts where lynching is a widespread tool in the repertoire of social control, can my theory on community ties account for its variation.

Second, the argument is centered on the social forces that facilitate collective action, but not on the motivation for the use of violence. It is thus complementary to existing literature on lynching. Frustration with the state (Jung and Cohen 2020) and moral outrage (Asif and Weenink 2022) are potential motivations for violence. The role of the state as weak, deficient, illegitimate, or even abusive is particularly important in this regard, as such factors provide opportunity and legitimacy to local communities to autonomously engage in social control (Taylor 1982). The acceptance of the practice of lynching and other forms of informal justice is directly related to the strength of the state (Dow et al. 2024; Krakowski and Kursani 2023). State agents may even negotiate with lynch mobs, tolerate them, and actively legitimate occasional violence for the purpose of social control (Fuentes Díaz and González 2022; Gaby et al. 2021). Also, a weak state may strengthen community ties, as community members try to compensate for the lack of public goods. In the below statistical analysis, I adjust for related alternative explanations where appropriate. In any case, my argument on community ties can best account for variation in lynching where state institutions have limited capacity.

## Research Strategy

### Mexico as Context

Contemporary Mexico is a suitable study context to examine my argument. First, lynching is a known and accepted tool in the repertoire of violence in many parts of Mexico. Lynching has been a historically common phenomenon, as Kloppe-Santamaría (2020) demonstrates in her study on lynching in the post-revolutionary period. She notes that the catalysts of lynching have changed, with witches and communists frequently targeted during her period of study (1930s to 1950s), and petty crime becoming more important over time. Today, lynching (the Spanish term is *linchamiento*) is not only omnipresent in the news, but the collective punishment of petty delinquents is often applauded in social media (Infobae 2020) and supported by large parts of society (CNDH 2019). The news media have historically played an important role in the construction of legitimating narratives and the presentation of lynching as acceptable, or even moral, behavior (Kloppe-Santamaría 2020:81; Piccato 2017).

According to the original lynching dataset created for this study (described below), Mexico is the country with the largest absolute number of reported lynching events in Latin America from 2010 to 2019, although Guatemala and Bolivia have higher rates. For the period from 2000 to February 2022, the dataset records a total of 1,859 lynching events in Mexico. The perhaps most emblematic case in this period is the lynching of three undercover police officers accused of child theft in a peripheral area of Mexico City in 2004. This case fueled outrage and was in the news for weeks due to the innocence of the officers, the extreme violence (two officers were burned to death), the live TV broadcast, and police inaction (Binford and Churchhill 2009). However, it is not representative of the modal case of lynching, which involves less severe violence and targets petty delinquents caught in flagrante.

**Figure 2.**
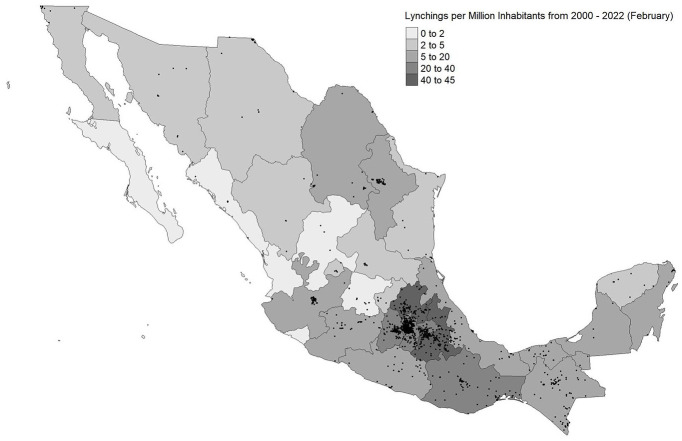
Lynchings across Mexican States

Figure 2 shows that lynching is concentrated in the central and southern areas of Mexico, including Mexico State, Mexico City, Puebla, Hidalgo, Morelos, Tlaxcala, Oaxaca, and Chiapas. Similar areas also saw the most lynchings from 1930 to 1959 (Kloppe-Santamaría 2020:127) and at the end of the twentieth century (Fuentes Díaz 2005), suggesting a continuity in the social structure underpinning violent collective action (see also Osorio, Schubiger, and Weintraub 2021). While lynching represents an accepted mode of social control, my argument indicates that variation in lynching events across the country depends on community ties, which may partially explain this geographic pattern.

Second, Mexico is a diverse country with a federalist state structure. Parts of the country have been historically weak in the administration of justice (Piccato 2017). Lynch mob participants interviewed by journalists often invoke their right to make justice on their own. This narrative resonates with Kloppe-Santamaría’s (2020) argument for lynching as a response to an illegitimate state, not an absent one. While regional differences are notable, there are high overall levels of impunity (95.7 percent of all homicides remained unresolved in 2022 [México Evalúa 2023]) and high levels of crime victimization. In 2021, 32 percent of Mexicans had been a victim of some crime within the previous 12 months (Lupu and Rodríguez 2021). Citizens often see crime reporting to authorities as useless and do not trust the government to provide justice. Roughly 93 percent of all crimes in 2022 were not reported (México Evalúa 2023). However, levels of impunity and victimization vary, with some areas in the Yucatán peninsula and parts of the north performing better. The “drug war” proclaimed in 2006 has differentially affected parts of the country (Magaloni et al. 2020; Trejo and Ley 2020), leading to diverse responses with regard to security. This includes a “move for security” to Mérida, which is seen as a safer city (Mattiace and Nonnenmacher 2022), and the formation of self-defense forces in the state of Michoacán (Moncada 2023).

Against the backdrop of widespread acceptance of lynching and regionally limited state capacity, my argument on community ties explains why some areas of Mexico experience more lynchings and why some individuals are more likely to participate. My analysis carefully addresses relevant sources of confounding, including variations in support for lynching across individuals and variations in state capacity across the Mexican territory.

### Analysis Strategy

I combine different empirical approaches (see Table 1). First, I focus on the relationship between community ties and lynching participation among individuals, using an original survey from Mexico City. I deal with confounding and examine the robustness and direction of the relationships, but some limitations remain. Second, I provide suggestive evidence based on the same original survey to study the mechanisms of solidarity and peer pressure. Third, going beyond individuals, I conduct aggregate-level analysis using cross-sectional municipality-level data covering the whole of Mexico. Fourth, to mitigate concerns about endogeneity, I created a municipality-year panel dataset, and I analyzed the effects of the 2017 Puebla earthquake, a plausibly exogenous shock for community ties. Following Shadish, Cook, and Campbell (2002:17), this shock can be interpreted as a “natural experiment.”

**Table 1.** Analysis Overview

Type of Analysis	Independent Variable	Dependent Variable	Level of Analysis	Geographic Scope
Individual-level analysis	Names known	Lynching participation	Individual	Mexico City
Analysis of mechanisms	Names known	Solidarity, peer pressure	Individual	Mexico City
Aggregate-level analysis	Neighborly cooperation	Lynching rate	Municipality (cross-section)	Republic of Mexico
Natural experiment	Earthquake exposure	Lynching events	Municipality (panel)	Republic of Mexico

A challenge for empirical analysis is that lynching can be a cause, and not only a consequence, of community ties (Bailey and Snedker 2011; Colombijn 2002; Fuentes Díaz 2005; Fujii 2021; Zizumbo-Colunga 2015). Participants may use the opportunity of a lynching to reinforce their ties and demonstrate a sense of unity (Nevels 2007). Because I am concerned here with the effect of community ties on lynching, the empirical analysis is designed to identify the causal arrow running from community ties to lynching.

As a general approach to the statistical procedures, I am interested in the direction of coefficients and whether they can be differentiated from zero with a high level of confidence, rather than in their specific magnitude. Also, I evaluate the coefficients in terms of consistency across modeling specifications; isolated “significant” results are considered less important.

## Individual-Level Analysis

### Data and Methods

In a first step, I examine the relationship between community ties and lynching participation across individuals surveyed in Mexico City. I chose Mexico City due to the prevalence of lynching (311 reported cases between 2000 and February 2022) and its administrative structure. The roughly 1,820 colonias provide a relatable small-scale unit for residents and are part of their home address. I use the colonias for sampling purposes and for analysis. Importantly, I can compare individuals within colonias (using fixed effects), thus keeping many contextual confounders constant across individuals.

In collaboration with the Mexican survey firm Data OPM, I conducted a face-to-face survey representative of the adult population of Mexico City in February 2022. Respondents were selected with a multi-stage sampling procedure (details in Part A1 of the online supplement). First, 340 colonias were identified using probability proportional to size sampling (Skinner 2016). Second, enumerators selected a random location and then used a random walk procedure, targeting six households in each colonia. This process resulted in a sample of 2,183 respondents (some colonias were oversampled). After extensive piloting, the questionnaire covered demographics, social behavior, security, and household surroundings, as well as questions about community ties and lynching (discussed below).

A survey about violence requires sound ethical considerations (details in A1 in the online supplement). Particular attention was dedicated to legal implications, psychological distress, safety risks, and data protection. I further ensured confidentiality, and I did not compensate participants financially. The university ethics board approved the procedures.

#### Dependent variable

The dependent variable is participation in lynching. I did not use the term “lynching” in the questionnaire, as it may evoke ambiguous associations, such as “social media lynching.” I instead anchored questions with a vignette describing a typical “mild” incident. The vignette said: “A thief assaults a lady on the street. Using a knife, he takes her belongings and escapes. After the robbery, a passer-by manages to take away the thief’s knife and subdues the thief. In this moment, a large number of people gather, insult, and punish the thief” (adapted from CNDH 2019).

The design of this vignette is consistent with the lynching dataset. In 59 percent of cases recorded in Mexico, mobs targeted a single person; in 95 percent of cases the targeted person was male; 65 percent of events occurred in response to alleged theft; and in 62 percent of cases, there was at least a collective beating, if not more severe violence (17 percent ended in fatality). The vignette presents the modal type of lynching in Mexico and is thus a valid representation.

The questionnaire asked about support and knowledge of events like the one described in the vignette. The question used in the below analysis asks whether respondents “have ever participated in an event like that,” including yes or no options. Among all respondents, 9.6 percent admitted having participated. Before this question, the survey inquired whether respondents “have ever witnessed an event like that” (yes: 23 percent). The survey context thus made it clear that participation goes beyond mere bystanding.

When asked about violence in surveys, people may give responses that are not truthful. The mild wording of the vignette should limit this possibility. Describing an extreme form of violence would have limited the reporting of truthful answers (Westwood et al. 2022). Following advice of a Mexican legal consultant, I decided to use a term (i.e., *castigar* [punish]) that does not describe a penal code entry, to avoid legal consequences for survey respondents.

The vignette presents a man assailant of a woman victim to make the presentation relatable. This common combination of male and female protagonists plays into prevalent gender norms that make it easier for respondents to support the protection of a vulnerable victim (Kreft and Agerberg 2024). Overall, 71.3 percent of respondents agreed with the neighbors who punished the thief, suggesting that social desirability bias is not a major concern, and supporting the above claim that community punishment of petty delinquents is a widely accepted practice. The reliability of the question about participation was assessed with three pilot surveys, which showed similar answer proportions (see Part A1 of the online supplement).

#### Independent variables

To capture community ties, I use a novel indicator created for this study. I asked respondents “how many people [they] know by name or nickname in their colonia” (mean: 36). Knowing someone’s name requires repeated previous personal interaction and is thus suggestive of a close social tie. Knowing more names means an individual has more connections to their community. This variable has the added advantage that it can be used as a meaningful number.^8^

Given the novelty of this indicator, I also use a more common indicator of community ties (community participation), drawn from the surveys of the *AmericasBarometer* by the Latin American Public Opinion Project (Lupu and Rodríguez 2021). Results are consistent (see Table A4.2 in the online supplement). Also, the principal component of two community participation indicators is correlated with knowing names in the colonia (coefficient: 0.15).

To capture interpersonal trust, I use the same indicator as used in the *AmericasBarometer*. On a 1 to 4 Likert scale, respondents were asked: “Talking about the people living in your neighborhood (Colonia) would you say they are . . . trustworthy?” (mean: 2.7). Interpersonal trust is weakly correlated with knowing names in the colonia (coefficient: 0.09).

### Analysis

I start with a linear regression analysis of community ties and lynching participation, adjusting for major sources of confounding, including a series of demographic characteristics that may be related to community ties, such as level of education, age, sex, socioeconomic standing, and employment status. I further adjust for past participation in a fight to account for individual propensity to violence; religious affiliation to account for views on justice and punishment (Bailey and Snedker 2011); trust in government to account for one’s view of state authority (Nivette 2016); number of siblings to account for differential childhood experiences and upbringing; whether one’s parents have lived in the same colonia to account for long-term connection to the locality; and commercial use and public space cleanliness of the street block to account for within-colonia differences related to social disorganization (Vilalta et al. 2020); the last two items were coded by enumerators in situ. Standard errors are clustered at the colonia level, which accounts for potential within-colonia correlation. One model further includes colonia-level fixed effects (keeping constant unobserved colonia-level confounders such as crime rate, state capacity, and location within the city).

Table 2 shows the results of this analysis (for the full table, see A4.1 in the online supplement), suggesting a positive relationship between community ties and lynching participation. Adjusting for an incremental number of covariates, the coefficient for names known remains very similar, even after including colonia fixed effects and increased model fit (*R*^2^). This suggests the relationship is not sensitive to omitted variable bias, in line with Oster’s (2019) procedures evaluating coefficient stability. As expected, interpersonal trust does not have the same relationship with lynching. Coefficients for trust are stable and negative in all models, although not always distinguishable from 0.

**Table 2.** Individual-Level Analysis: Community Ties and Lynching Participation

	(1)	(2)	(3)	(4)
Names known (log)	.036*** (.006)	.033*** (.006)	.027*** (.007)	.032*** (.008)
Trust	−.018* (.008)	−.019* (.008)	−.018* (.008)	−.017 (.010)
Constant	.040 (.029)	.088* (.039)	.082 (.052)	−.067 (.063)
*N*	2,129	2,117	2,060	2,060
Adj. *R*^2^	.019	.028	.065	.081
Colonia FE	No	No	No	Yes
Control variables	No	Some	All	All

*Note*: Standard errors are in parentheses. OLS regression, standard errors clustered at colonia level (340 clusters). Varying *N* due to non-response.

* *p* < 0.05; **p < 0.01; ***p < 0.001 (two-tailed test).

**Figure 3.**
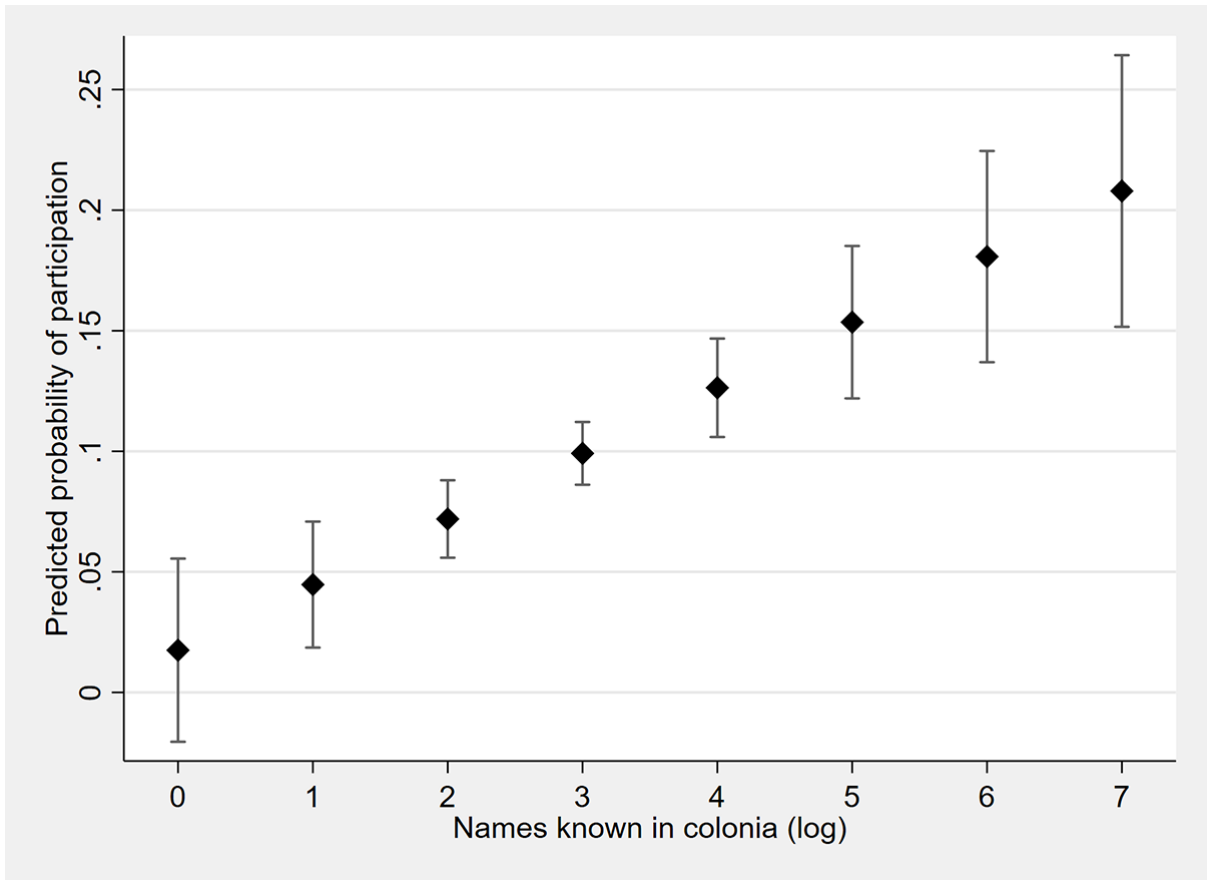
Predictive Margins with 95 Percent Confidence Intervals

Due to the logged independent variable and a binary outcome variable, the relationship between community ties and lynching is difficult to interpret. Figure 3 shows the predicted margins for Model 3 of Table 2, which indicates a continuous relationship between names known in the colonia (logged) and the predicted probability of lynching participation.

Tables A4.2, A4.3, and A4.4 in the online supplement show that results are robust under the following conditions: using a classical indicator of social ties (community participation), using logistic instead of linear regression, and excluding outliers in terms of names known. Having the opportunity to participate in a lynching is a major source of confounding in this set-up. In an additional analysis, I therefore also adjust for whether an individual has witnessed a lynching, which allows us to compare individuals who effectively had a chance to participate. Coefficients remain stable, and overall model fit increases, suggesting again that omitted variable bias is limited (see Table A4.5 in the online supplement). Restricting the sample to only individuals who witnessed a lynching (*N* = 498), the relationship between ties and participation remains robust (Table A4.6). The same holds when accounting for whether individuals agree with lynching (Table A4.7) and accounting for previous lynchings in the colonia (Table A4.8).

Although I account for sources of confounding, community ties can be either a cause or a consequence of lynching participation in this cross-sectional set-up. To mitigate concerns about reverse causation, I use a temporally prior proxy for community ties: parents’ number of siblings (i.e., number of aunts and uncles). The rationale for use of this proxy is the following. Community ties depend on one’s own efforts to socialize and the social connections received at birth. Parents’ number of siblings is related to the latter and potentially increases the number of community ties, as, all else being equal, more family connections mean more relationships through them. However, all else may not be equal, as the number of aunts and uncles may introduce unobserved bias: poorer and less educated grandparents tend to have more children, which may have a knock-on effect on subsequent generations. To account for unobserved imbalances, I adjust for the number of a respondent’s own siblings, which should account for the intergenerational knock-on effect, if the conditions leading to more children did not change between the grandparents’ and parents’ generation. I thereby effectively “control for a descendant,” which shuts the backdoor path from the grandparents’ unobserved life conditions to lynching participation (Pearl and Mackenzie 2018:158). Table A4.9 in the online supplement suggests that having more aunts and uncles is related to a higher likelihood of lynching participation, even after adjusting for own siblings, providing plausible evidence for a causal arrow pointing from community ties to lynching participation.

## Mechanisms

### Data and Methods

I have argued that community ties operate through solidarity and peer pressure. This implies that individuals who are more heavily tied to their communities should be more exposed to these social forces. I therefore analyze the relationship between community ties (as well as trust) and indicators of these mechanisms.

I use indicative questions from the same survey in Mexico City to examine this implication (response options were measured on a 1 to 4 Likert scale). For solidarity, respondents were asked whether they “feel united with the neighbors of their colonia” (53 percent said they feel quite or very united). Individuals with more community ties should feel more united with their neighbors.

For peer pressure, respondents were asked whether they think “their neighbors would encourage them to join the [vignette-described lynching] event” (43 percent responded affirmatively). Individuals with more community ties should be more likely to say their neighbors would push them to participate.

The two indicators are related to participation in lynching in the expected direction. The below analysis focuses on their relationship to community ties, a necessary condition for their relevance as mechanisms.

### Analysis

Table 3 shows results using the same modeling specifications as Table 2 (Models 2 and 4, for the full table, see Table A4.10 in the online supplement). As expected, community ties are positively related to feelings of solidarity and neighborly peer pressure. Interpersonal trust is related to solidarity but not peer pressure, suggesting again a difference between the consequences of community ties and trust. Taken together, community ties likely act on lynching participation through the posited mechanisms, specifically through peer pressure.

**Table 3.** Mechanisms: Solidarity and Peer Pressure

	(1)	(2)	(3)	(4)
	United with Neighbors	United with Neighbors	Neighbors Encourage Lynching	Neighbors Encourage Lynching
Names known (log)	.197*** (.017)	.203*** (.021)	.126*** (.022)	.098*** (.029)
Trust	.327*** (.025)	.306*** (.031)	−.048 (.030)	−.025 (.035)
Constant	.966*** (.103)	.789*** (.186)	2.426*** (.139)	2.137*** (.247)
*N*	2,114	2,057	2,047	1,993
Adj. *R*^2^	.166	.189	.047	.088
Colonia FE	No	Yes	No	Yes
Control variables	Some	All	Some	All

*Note*: Standard errors are in parentheses. OLS regression, standard errors clustered at colonia level (340 clusters). Varying *N* due to non-response.

* *p* < 0.05; **p < 0.01; ***p < 0.001 (two-tailed test).

In the online supplement, I examine whether community ties are related to alternative mechanisms for participation in lynching. Previous literature indicates that lynching depends on crime threat perceptions (Gamallo 2020; Godoy 2006), trust in government (Jung and Cohen 2020; Smith 2019), and collectivist moral values (Asif and Weenink 2022; Nussio 2023). These explanations of lynching can only account for the relationship between community ties and lynching participation if they are also related to community ties. Analysis suggests this is not the case (see A4.11 in the online supplement). Community ties are thus a separate driver of lynching independent from prior explanations.

## Aggregate-Level Analysis

### Data and Methods

To expand the analysis to the aggregate level and to a wider context, I created a cross-sectional municipality dataset covering the whole of Mexico.

#### Dependent variable

To capture lynching, I use the original lynching in Latin America dataset (see Part A2 of the online supplement). This dataset was created by a research team who coded news reports of lynching incidents (see Nussio and Clayton 2024).^9^ Events are geo-coded and aggregated to the municipality level. Given the skewed distribution of lynching events across municipalities, I use the log of lynching per million inhabitants in the main analysis. Population data are based on census information recorded by the National Statistics Office (INEGI).

Lynching event data are affected by reporting and urban bias, so my analysis strategy addresses these important concerns. Data collection procedures, biases, and comparisons to other datasets on lynching in Mexico are described in Part A2 of the online supplement. The lynching dataset used in this study reports similar frequencies of lynching over time and across space as data collected by other researchers (e.g., CNDH 2019; Rodríguez Guillén and Veloz Ávila 2019).

#### Independent variables

Municipality-level data on community ties are not readily available. Therefore, I draw on high-quality, mass-scale surveys conducted by the INEGI: the Encuesta Nacional de Victimización y Percepción sobre Seguridad Pública (ENVIPE). The yearly ENVIPE surveys use multi-stage random sampling. All respondents from ENVIPE surveys conducted from 2011 to 2020 (*N* = 854,720) were aggregated to their respective municipality of residence to calculate average municipality characteristics. This aggregation is an adequate approach as the ENVIPE surveys are based on random sampling within municipalities. By pooling 10 years of surveys, I reduce random measurement error. I checked how closely the ENVIPE data correspond to census information using the variable on education level, which is measured by both. The ENVIPE municipality aggregate variable of education level (the mean survey-reported level of education) has a high correlation with the census variable (0.87). Using only ENVIPE municipality aggregates with larger numbers of observations produces even higher levels of correlation, suggesting the larger the number of individual survey respondents to calculate each municipality observation, the less measurement error (see also Jones and Norrander 1996). I therefore conducted sensitivity analyses that consider the number of survey participants used for the calculation of each municipality observation, and results remain substantively the same. Potential sources of bias are described further in Part A3 of the online supplement.

To capture community ties, I use a question about neighborly cooperation, which reflects the aspect of community ties and community-level reciprocity described by Taylor (1982): whether most neighbors helped each other resolve common interest problems. Neighborly cooperation is a result of community ties, rather than constitutive of them, a common relationship between basic concept and indicator (Goertz 2006:58). I am unaware of a more suitable variable capturing aggregate-level community ties in Mexico with better geographic coverage. The variable corresponds to the proportion of affirmative answers (mean: 0.48). The problems referred to in the questionnaire are related to lighting, water, and water leakages. I mainly draw on the first option (lighting), as it was the most often mentioned problem affecting a community. I also use water and water leakage problems, and a variable derived from principal component analysis (using all three) in robustness analyses. The main question was included in all surveys from 2012 to 2020 and covers more than 1,500 of the 2,457 municipalities. One limitation of this variable relates to the fact that problems with public infrastructure may be less common in wealthier areas; I therefore adjust for socioeconomic conditions.

For trust, I use a standard question about interpersonal trust contained in the ENVIPE surveys. I use the proportion of respondents who say they trust their neighbors (mean: 0.56). Interpersonal trust is weakly negatively correlated with neighborly cooperation on the municipality level (coefficient: –0.07).

### Analysis

I examine the relationship between neighborly cooperation as an indicator of community ties and the natural log of lynching per million inhabitants using linear regression analysis. I adjust for different sources of confounding (see Part A3 in the online supplement): whether there is a problem with lighting; municipality characteristics associated with urbanity, including population size and surface area; levels of poverty and inequality, as neighborly cooperation for a public good may be more common in poorer neighborhoods; the proportion of indigenous populations, given they may have particularly abundant community ties; the proportion of people who claim to be non-religious, thereby accounting for individualistic values; and officially reported robberies and homicide, as well as self-reported victimization, as these may affect community ties. In addition, I cluster standard errors at the *Estado* level to account for potentially clustered community practices (Abadie et al. 2017). In some specifications, I instead include Estado fixed effects to account for unobserved sources of confounding at the Estado level, such as geographic location, climate conditions, and state-level security practices (Mexico is a federal state).

Table 4 shows the main results, suggesting a positive relationship between neighborly cooperation and lynching rate (for the full table, see Table A4.12 in the online supplement). Adjusting for an incremental number of covariates and with increasing model fit, the coefficients of neighborly cooperation remain between 2.1 and 2.6. In Model 4, the coefficient is smaller due to the inclusion of Estado fixed effects. The size of the coefficients cannot be directly interpreted, as the dependent variable is the log of lynching per million. Using the lynching rate on its original scale, we have a coefficient of 41. This means that for every 0.1 increase in the proportion of neighborly cooperation (e.g., from the mean of 0.48 to 0.58), a municipality has 4.1 more lynchings per million inhabitants (mean is 18.6). Interpersonal trust is negatively related to lynching rate, although not all coefficients are distinguishable from 0. These results again suggest the relationship to lynching is specific to community ties and not to any kind of social capital.

**Table 4.** Aggregate-Level Analysis: Community Ties and Lynching Rate

	(1)	(2)	(3)	(4)
Neighborly cooperation	2.126** (.601)	2.538*** (.572)	2.178*** (.580)	1.310*** (.382)
Trust	−1.901* (.729)	−1.954** (.677)	−1.014 (.509)	−.271 (.389)
Constant	1.001 (.572)	1.316 (.848)	.638 (1.052)	−.680 (.948)
*N*	1,589	1,589	1,557	1,557
Adj. *R*^2^	.044	.112	.169	.269
Control variables	No	Some	All	All
Estado FE	No	No	No	Yes
Estado clustered SE	Yes	Yes	Yes	No

*Note*: Standard errors are in parentheses. Linear regression with cross-sectional municipality data and different specifications.

* *p* < 0.05; **p < 0.01; ***p < 0.001 (two-tailed test).

In Tables A4.13 and A4.14 in the online supplement, I use a “non-equivalent outcome” logic (Shadish et al. 2002). Non-equivalent outcomes must be similar to lynching but should not be related to community ties. I focus on homicide and robbery rates and find that community ties are not related to them, suggesting my findings are specific to lynching and not violence in general. In addition, findings are robust under the following conditions (see Tables A4.15 to A4.18 in the online supplement): using principal component analysis to create an indicator of neighborly cooperation, using Poisson instead of linear regression (without logging the dependent variable), including only lynchings with a reported fatality or injury, and including only fatal lynchings (corresponding to the definition of lynching often used in the United States). Given that the independent variable (neighborly cooperation) is measured at the individual level using ENVIPE surveys and aggregated to the municipality level, this variable is exposed to random measurement error. This error depends on the number of individual observations per municipality. I therefore ran additional models including only municipalities with at least 50, 100, and 300 individual-level observations per municipality. Results remain robust (see Tables A4.19 to A4.21 in the online supplement).

## Natural Experiment

### Data and Methods

To alleviate concerns about endogeneity, I use a plausibly exogenous source of variation for community ties: exposure to a natural disaster. Previous research shows natural disasters can strengthen social ties between community members (Calo-Blanco et al. 2017; Solnit 2010; Whitt and Wilson 2007; Yamamura 2016), although this is not always the case (Fleming, Chong, and Bejarano 2014).

I study the effect of the largest natural disaster in Mexico in recent years, the Puebla earthquake on September 19, 2017, which affected the central and south-central area of Mexico, killing 371 people (mostly in Mexico City) and injuring another 3,289 (mostly in Morelos) (Instituto Belisario Domínguez 2017). Ample anecdotal evidence suggests this earthquake was a shock to community ties, increasing mutual aid and solidarity in its aftermath, with a wealth of pictures of neighbors cooperating in the search for survivors and clearing the rubble (BBC News Mundo 2017). Many observers compared the spontaneous acts of solidarity with what happened after the devastating earthquake in 1985 on the exact same date (Allier Montaño 2018). The disaster thus provides a plausibly exogenous source of variation for local community ties. According to my argument, the earthquake should be followed by increased levels of lynching in areas exposed to the event.

To assess this observable implication, I created a municipality-year panel dataset and used a two-way fixed-effects approach (Cunningham 2021). The dependent variable for this analysis is the number of lynching events. The key independent variable is an interaction term of earthquake shock (before versus after) and earthquake exposure (geographically exposed or not). The earthquake happened on September 19, 2017. Hence, I code the year 2018 and later as 1 and otherwise 0 (for other specifications, see the online supplement). To capture exposure, I use three variables: municipalities within a 250-kilometer radius of the earthquake, municipalities that belong to states with some level of damage from the earthquake, and distance from the earthquake.

The main limitation is that Mexico is a disaster-prone country, with recurrent earthquakes, floodings, and hurricanes (Alcántara-Ayala 2019). Some of these disasters may have overlapped with the 2017 earthquake (I replicated the analysis focusing on a shorter time window). Also, the earthquake of 2017 affected areas of Mexico where citizens already had low levels of trust in the state. This may have amplified the effect, as community ties increased where a source of motivation to perpetrate lynchings was present.

### Analysis

In the below analysis, I adjust for covariates that vary across units and over time, privileging variables with high panel coverage, including infant mortality, homicides, and robberies. Variables that vary only across units or only over time are absorbed by the two-way fixed effects. Standard errors are clustered at the municipality level due to autocorrelation.

Table 5 shows the results (for the full table, see Table A4.22 in the online supplement). For all three specifications, earthquake exposure is related to lynching events (models with control variables cover 2012 to 2019). In Models 5 and 6, coefficients are negative as the interaction term captures the distance from the earthquake (with higher distance, fewer lynchings). Coefficients generally remain in the same range with and without control variables. The coefficient from Model 2 means areas exposed to the 2017 earthquake experienced an average increase of 0.11 lynching events per year compared to municipalities that were not exposed, a substantively small effect. The mean of yearly lynching events per municipality is 0.034, due to the rarity of lynchings in small municipalities. When restricting the analysis to the 40 largest municipalities, the coefficient is 3.2 (see Table A4.24 in the online supplement).

**Table 5.** Natural Experiment: Earthquake Exposure and Lynching

	(1)	(2)	(3)	(4)	(5)	(6)
Within 250km from earthquake × After 2017	.108*** (.019)	.113*** (.021)				
Earthquake damage × After 2017			.087*** (.019)	.126*** (.026)		
Distance from earthquake in 100km × After 2017					−.010*** (.002)	−.009*** (.002)
Constant	.008* (.003)	.073* (.035)	.008* (.003)	.069* (.034)	.008* (.003)	.075* (.035)
*N*	51,597	17,225	51,597	17,225	51,597	17,225
Adj. *R*^2^	.017	.065	.015	.066	.014	.062
Control variables	No	Yes	No	Yes	No	Yes

*Note*: Standard errors are in parentheses. Linear regression with year and municipality fixed effects, and municipality clustered standard errors.

* *p* < 0.05; **p < 0.01; ***p < 0.001 (two-tailed test).

In the online supplement, I examine the robustness of these findings (see A4.23 to A4.31). Given that lynching event data are based on news reports, I first check whether the earthquake led to differential newspaper reporting, which is not the case (procedures described in A4.23). Results are consistent when adjusting for total news reporting, and with the inclusion of a lagged dependent variable, when dropping the year 2017 (during which the earthquake happened), when restricting the analysis to only 2016 to 2018 (which reduces the influence of other occurrences), when dropping any one of the three most influential states from the analysis (Mexico City, Mexico State, or Puebla), when including only lynchings that ended in fatality, and when using Poisson instead of linear regression. One may argue that the earthquake increased lynchings due to a general increase in violence and crime. To account for that, I adjust for levels of crime in all the displayed models. In addition, I use non-equivalent outcomes, which provide mixed results: homicides are negatively related to earthquake exposure and robberies positively related (but less strongly than lynching). A generalized turn to violence thus does not explain increased lynchings. Also, an increase in crime due to post-earthquake looting would only concern the first few days and would not explain the encountered pattern. These robustness tests are particularly important as lynchings seemed to increase in the years before the earthquake in and around Mexico City. However, the interaction term of exposure and time of earthquake remains largely robust, even in the short time window of 2016 to 2018. Hence, an earlier trend toward an increase seems to be amplified by the earthquake.

One limitation of the natural experiment is the lack of a time-varying measure of community ties. I therefore cannot be sure that earthquake exposure affects lynching through increased community ties.^10^ To mitigate this limitation, I used another measure for disaster exposure in the online supplement: exposure to active volcanos. The results of cross-sectional analysis suggest municipalities in closer proximity to volcanos have both more community ties and higher lynching rates (see A4.32 in the online supplement).

## Conclusions

In this article, I argued that community ties help overcome the high barriers for violent collective action. Analyses based on evidence from individuals living in Mexico City and Mexican municipalities show community ties facilitate lynching, a common form of violent collective action around the globe. I present suggestive evidence that the mechanisms of solidarity and peer pressure influence individuals. A natural experiment and additional analyses allow for a cautious causal interpretation of the relationship between community ties and lynching. While each separate empirical approach is limited, the combined wealth of new evidence presented in this study provides consistent support for the argument.

These findings have important implications for theories on social structure and group behavior. Fears about the negative effects of community ties are justified, as Putnam (2002:11) recognized when he warned that communities with excessive bonding social capital may pursue “sinister ends.” Several authors writing on lynching have warned about the dangers of tightly-knit communities without systematically examining their suspicion (Godoy 2006; Goldstein et al. 2007). Previous studies have shown aggregate-level connections between bonding social capital and collective violence, including terrorism, gangs, and genocide (Alcorta et al. 2020; Krakowski 2021; McDoom 2014), but the present study provides robust evidence. Lynching often involves perpetrators who reside in the same location, making it an ideal form of violence to examine the “dark side” of community ties. With newly created data, I not only show an aggregate-level relationship, but also that community ties drive individual participation in lynchings—an original contribution of this article.

This study has implications for collective efficacy theory, which claims that neighborly social cohesion (in addition to shared normative expectations) is a driver of effective social control and crime prevention (Sampson 2008). In a paradoxical way, my findings on lynching lend support to this theory, even though lynching is itself a crime. The apparent contradiction is resolved when considering that lynching is not only a crime, but also a form of social control. After all, lynching punishes wrongdoing, a threat to shared community norms (Black 1997; Garland 2005; Mead 1918; Senechal de la Roche 1996). From the perpetrators’ perspective, lynching contributes to the public goods of retributive justice and deterrence. However, the actual effectiveness of lynch mobs for preventing delinquency is largely unknown. From the state’s perspective, lynching creates the public bad of insecurity. It is from this latter perspective that community ties reveal a “dark side.”

This study also has implications for other forms of collective violence that we usually see through the lens of grievances and identity theory, like ethnic conflict and riots (Cederman, Gleditsch, and Buhaug 2013; Horowitz 2001). These forms of violence also draw on community ties. In fact, “*catnet*”—the combination of category (or identity group) and network (or social ties)—may be most mobilizing, as Tilly (1978) suggested. In contrast, the lack of community ties in neighborhoods of atomized societies may prevent locally based collective violence. Tellingly, collective violence in such societies is often related to subcultures that foster strong connections beyond the confines of local community, like hooliganism (Dunning, Murphy, and Williams 1988) and jihadism (Cottee 2011).

Latin America is the world region with the highest rates of everyday violence (UNODC 2019). Lynching may be seen as less important compared to urgent challenges like drug cartels in Mexico, armed conflict in Colombia, political turmoil in Venezuela, and the expansion of criminal governance across several countries (Lessing 2021). Policymakers thus rarely focus on lynching, even though it is clearly related to state deficiencies and undermines state legitimacy. What can they learn from the present article? A naïve reading may lead to the conclusion that the prevention of lynching requires the destruction of community ties. This should not be the key takeaway. Rather, policymakers should focus on the part of the game they can control: strengthening their authority and increasing trust among community members. Government actors can complement excessive bonding within communities, which as I show facilitates violent social control, with bridging toward state agents, thereby reducing support for lynching (Nivette 2016). Policies like community policing (Gill et al. 2014) and initiatives improving procedural justice (Karim 2020; Wood, Tyler, and Papachristos 2020) may contribute to trust relationships, but they need to be adapted to the Latin American context (Blattman et al. 2022; González and Mayka 2023; Montambeault and Dias Félix 2021). Such policies could strengthen the norms shared by community members and state agents, enabling a concerted engagement in social control and bringing the “bright side” of community ties to the fore.

## Supplemental Material

sj-pdf-1-asr-10.1177_00031224241253268 – Supplemental material for The “Dark Side” of Community Ties: Collective Action and Lynching in Mexico Supplemental material, sj-pdf-1-asr-10.1177_00031224241253268 for The “Dark Side” of Community Ties: Collective Action and Lynching in Mexico by Enzo Nussio in American Sociological Review

## Notes

1. Although I agree with criticism of Olson’s theory (e.g., Marwell and Oliver 1993), his specification of the collective action problem remains a useful starting point.

2. In my analysis, I also use an operationalization of lynching including fatal outcome. Results remain substantively similar.

3. However, lynching can be embedded in wider organizational dynamics. Smångs (2016) describes how the activities of the Democratic Party in the U.S. South fomented the racial boundaries and in-group solidarity that facilitated lynching.

4. Several authors use the term vigilantism similarly to my understanding of lynching (e.g., Dow et al. 2024; Smith 2019).

5. In the case of punishment like lynching (Garland 2005), participating as punisher is costly and the benefits of punishment are nonexcludable (Elster 1989:40).

6. Taylor argues that communities are further characterized by shared values, an aspect that is relevant for lynching but goes beyond the scope of this article (see Nussio 2023).

7. Similar to Taylor’s view of community including shared values, Sampson (2008:154) argues that collective efficacy requires shared expectations in addition to social connectivity.

8. I abstained from inquiring about specific names, as respondents might have perceived that as intrusive.

9. To download the dataset and supporting materials, see https://css.ethz.ch/en/research/datasets/lynching-in-latin-america.html.

10. The ENVIPE survey data do not allow for the construction of time-varying municipality-year measures (see A4.22 in the online supplement).

## References

[bibr1-00031224241253268] Abadie Alberto Athey Susan Imbens Guido W. Wooldridge Jeffrey . 2017. “When Should You Adjust Standard Errors for Clustering?” Working Paper 24003, National Bureau of Economic Research.

[bibr2-00031224241253268] Agostini Maximilian van Zomeren Martijn . 2021. “Toward a Comprehensive and Potentially Cross-Cultural Model of Why People Engage in Collective Action: A Quantitative Research Synthesis of Four Motivations and Structural Constraints.” Psychological Bulletin 147(7):667–700. 34855427 10.1037/bul0000256

[bibr3-00031224241253268] Alcántara-Ayala Irasema . 2019. “Desastres En México: Mapas y Apuntes Sobre Una Historia Inconclusa.” Investigaciones Geográficas 100 (10.14350/rig.60025).

[bibr4-00031224241253268] Alcorta Ludovico Smits Jeroen Swedlund Haley J. de Jong Eelke . 2020. “The ‘Dark Side’ of Social Capital: A Cross-National Examination of the Relationship between Social Capital and Violence in Africa.” Social Indicators Research 149(2):445–65.

[bibr5-00031224241253268] Allier Montaño Eugenia . 2018. “Memorias Imbricadas: Terremotos En México, 1985 y 2017.” Revista Mexicana de Sociología 80(spe):9–40.

[bibr6-00031224241253268] Asif Muhammad Weenink Don . 2022. “Vigilante Rituals Theory: A Cultural Explanation of Vigilante Violence.” European Journal of Criminology 19(2):163–82.

[bibr7-00031224241253268] Bailey Amy Kate Snedker Karen A. . 2011. “Practicing What They Preach? Lynching and Religion in the American South, 1890–1929.” American Journal of Sociology 117(3):844–87. 10.1086/661985 PMC3856205 24327771

[bibr8-00031224241253268] Bailey Amy Kate Tolnay Stewart E. . 2015. Lynched: The Victims of Southern Mob Violence. Chapel Hill: University of North Carolina Press.

[bibr9-00031224241253268] Ball Joanna . 1994. The Ritual of the Necklace. Cape Town: Centre for the Study of Violence and Reconciliation.

[bibr10-00031224241253268] Bandura Albert . 2016. Moral Disengagement: How People Do Harm and Live with Themselves. New York: Worth.

[bibr11-00031224241253268] Barolsky Vanessa . 2016. “‘Loving Thy Neighbour’ in Times of Violence: Social Cohesion and Collective Efficacy in South Africa.” Psychology in Society 51:1–27 (http://dx.doi.org/10.17159/2309-8708/2016/n51a1).

[bibr12-00031224241253268] Bateson Regina. 2013. “Order and Violence in Postwar Guatemala.” PhD thesis, Yale University, New Haven, CT.

[bibr13-00031224241253268] Bateson Regina . 2021. “The Politics of Vigilantism.” Comparative Political Studies 54(6):923–55.

[bibr14-00031224241253268] BBC News Mundo. 2017. “‘Las lágrimas se me salían sin parar al ver tanta ayuda y oír a la gente cantar’: la solidaridad en México tras el terremoto.” September 20.

[bibr15-00031224241253268] Beck E. M. Tolnay Stewart E. Bailey Amy Kate . 2016. “Contested Terrain: The State versus Threatened Lynch Mob Violence.” American Journal of Sociology 121(6):1856–84.

[bibr16-00031224241253268] Benevides Maria-Victoria Fischer Rosa-Maria . 1991. “Popular Responses and Urban Violence: Lynching in Brazil.” Pp. 33–45 in Vigilantism and the State in Modern Latin America, edited by Huggins M. K. . New York: Greenwood Publishing Group.

[bibr17-00031224241253268] Berg Manfred Wendt Simon . 2011. “Introduction: Lynching from an International Perspective.” Pp. 1–18 in Globalizing Lynching History. New York: Palgrave Macmillan.

[bibr18-00031224241253268] Bicchieri Cristina . 2002. The Grammar of Society: The Nature and Dynamics of Social Norms. New York: Cambridge University Press.

[bibr19-00031224241253268] Binford Leigh Churchhill Nancy . 2009. “Lynching and States of Fear in Urban Mexico.” Anthropologica 51(2):301–12.

[bibr20-00031224241253268] Black Donald . 1997. The Social Structure of Right and Wrong, rev. ed. San Diego, CA: Emerald.

[bibr21-00031224241253268] Blattman Christopher Duncan Gustavo Lessing Benjamin Tobon Santiago . 2022. “Civilian Alternatives to Policing: Evidence from Medellín’s Community Problem-Solving Intervention Operación Convivencia.” National Bureau of Economic Research.

[bibr22-00031224241253268] Brundage William Fitzhugh . 1993. Lynching in the New South: Georgia and Virginia, 1880–1930. Chicago: University of Illinois Press.

[bibr23-00031224241253268] Bulutgil H. Zeynep Prasad Neeraj . 2023. “Inequality, Elections, and Communal Riots in India.” Journal of Peace Research 60(4):619–33.

[bibr24-00031224241253268] Calo-Blanco Aitor Kovářík Jaromír Mengel Friederike Romero José Gabriel . 2017. “Natural Disasters and Indicators of Social Cohesion.” PLOS ONE 12(6):e0176885 (10.1371/journal.pone.0176885). PMC5462365 28591148

[bibr25-00031224241253268] Castillo Claudett Eduardo . 2000. “La justicia en tiempos de la ira: linchamientos populares urbanos en América Latina.” Ecuador Debate 51:207–26.

[bibr26-00031224241253268] Cederman Lars-Erik Gleditsch Kristian Skrede Buhaug Halvard . 2013. Inequality, Grievances, and Civil War. New York: Cambridge University Press.

[bibr27-00031224241253268] CNDH. 2019. Informe Especial Sobre Los Linchamientos En México. Mexico: Comisión Nacional de Derechos Humanos.

[bibr28-00031224241253268] Coleman James S. 1988. “Social Capital in the Creation of Human Capital.” American Journal of Sociology 94:S95–S120.

[bibr29-00031224241253268] Coleman James S. 1990. Foundations of Social Theory. Boston: Harvard University Press.

[bibr30-00031224241253268] Collins Randall . 2008. Violence: A Micro-Sociological Theory. Princeton, NJ: Princeton University Press.

[bibr31-00031224241253268] Colombijn Freek . 2002. “Maling, Maling! The Lynching of Petty Criminals.” Pp. 299–330 in Roots of Violence in Indonesia, edited by Colombijn F. Lindblad T. . Leiden, Netherlands: Brill.

[bibr32-00031224241253268] Cooney Mark . 1998. “The Dark Side of Community: Moralistic Homicide and Strong Social Ties.” Sociological Focus 31(2):135–53.

[bibr33-00031224241253268] Costalli Stefano Ruggeri Andrea . 2015. “Indignation, Ideologies, and Armed Mobilization: Civil War in Italy, 1943–45.” International Security 40(2):119–57.

[bibr34-00031224241253268] Cottee Simon . 2011. “Jihadism as a Subcultural Response to Social Strain: Extending Marc Sageman’s ‘Bunch of Guys’ Thesis.” Terrorism and Political Violence 23(5):730–51.

[bibr35-00031224241253268] Cruz José Miguel Kloppe-Santamaría Gema . 2019. “Determinants of Support for Extralegal Violence in Latin America and the Caribbean.” Latin American Research Review 54(1):50–68.

[bibr36-00031224241253268] Cunningham Scott . 2021. Causal Inference: The Mixtape. New Haven, CT: Yale University Press.

[bibr37-00031224241253268] Cushman Fiery Gray Kurt Gaffey Allison Mendes Wendy Berry . 2012. “Simulating Murder: The Aversion to Harmful Action.” Emotion 12(1):2–7. 21910540 10.1037/a0025071

[bibr38-00031224241253268] della Porta Donatella . 1995. Social Movements, Political Violence, and the State: A Comparative Analysis of Italy and Germany. Cambridge, UK: Cambridge University Press.

[bibr39-00031224241253268] Dow David A. Levy Gabriella Romero Diego Tellez Juan Fernando . 2024. “State Absence, Vengeance, and the Logic of Vigilantism in Guatemala.” Comparative Political Studies 57(1):147–81.

[bibr40-00031224241253268] Dunning Eric Murphy Patrick J. Williams John . 1988. The Roots of Football Hooliganism: An Historical and Sociological Study. London, UK: Routledge.

[bibr41-00031224241253268] Durkheim Emile . 1893. The Division of Labor in Society. New York: Simon and Schuster.

[bibr42-00031224241253268] Elster Jon . 1989. The Cement of Society: A Survey of Social Order. Cambridge, UK: Cambridge University Press.

[bibr43-00031224241253268] Elster Jon . 2007. Explaining Social Behavior: More Nuts and Bolts for the Social Sciences. Cambridge, UK: Cambridge University Press.

[bibr44-00031224241253268] El Universal. 2019. “En una mañana, dos intentos de linchamiento.” San Luis Potosí, March 28.

[bibr45-00031224241253268] Epperly Brad Witko Christopher Strickler Ryan White Paul . 2020. “Rule by Violence, Rule by Law: Lynching, Jim Crow, and the Continuing Evolution of Voter Suppression in the U.S.” Perspectives on Politics 18(3):756–69.

[bibr46-00031224241253268] Eriksson Kimmo Strimling Pontus Gelfand Michele . 2021. “Perceptions of the Appropriate Response to Norm Violation in 57 Societies.” Nature Communications 12(1):1481. 10.1038/s41467-021-21602-9 PMC7935962 33674587

[bibr47-00031224241253268] Fleming David A. Chong Alberto Bejarano Hernán D. . 2014. “Trust and Reciprocity in the Aftermath of Natural Disasters.” The Journal of Development Studies 50(11):1482–93.

[bibr48-00031224241253268] Freire Danilo Skarbek David . 2023. “Vigilantism and Institutions: Understanding Attitudes toward Lynching in Brazil.” Research & Politics 10(1) (10.1177/20531680221150389).

[bibr49-00031224241253268] Fuentes Díaz Antonio . 2005. “El Estado y La Furia.” El Cotidiano 131:7–19.

[bibr50-00031224241253268] Fuentes Díaz Antonio González José Alberto . 2022. “De La Vigilancia al Vigilantismo. El Caso de Los Linchamientos Perpetrados Por Colectivos Vecinales En Puebla.” Pp. 141–66 in Vigilantismo en América Latina: Violencias colectivas, apropiaciones de la justicia y desafíos a la seguridad pública, edited by Fuentes Díaz A. Gamallo L. Quiroz L. . Buenos Aires: CLACSO.

[bibr51-00031224241253268] Fujii Lee Ann . 2021. Show Time: The Logic and Power of Violent Display. Ithaca, NY: Cornell University Press.

[bibr52-00031224241253268] Gaby Sarah Cunningham David Lee Hedwig Ward Geoff Jackson Ashley N. . 2021. “Exculpating Injustice: Coroner Constructions of White Innocence in the Postbellum South.” Socius 7 (10.1177/2378023120983647).

[bibr53-00031224241253268] Gamallo Leandro . 2015. “Los Linchamientos En México En El Siglo XXI.” Revista Mexicana de Sociología 77(2):183–213.

[bibr54-00031224241253268] Gamallo Leandro . 2020. De La Furia a La Acción Colectiva: Las Represalias Violentas En Argentina. Buenos Aires: Peter Lang.

[bibr55-00031224241253268] Garland David . 1990. Punishment and Modern Society: A Study In Social Theory. Chicago: University of Chicago Press.

[bibr56-00031224241253268] Garland David . 2005. “Penal Excess and Surplus Meaning: Public Torture Lynchings in Twentieth-Century America.” Law & Society Review 39(4):793–834.

[bibr57-00031224241253268] Giardini Francesca Wittek Rafael . 2019. “Gossip, Reputation, and Sustainable Cooperation.” In The Oxford Handbook of Gossip and Reputation. Oxford, UK: Oxford University Press.

[bibr58-00031224241253268] Gill Charlotte Weisburd David Telep Cody W. Vitter Zoe Bennett Trevor . 2014. “Community-Oriented Policing to Reduce Crime, Disorder and Fear and Increase Satisfaction and Legitimacy among Citizens: A Systematic Review.” Journal of Experimental Criminology 10(4):399–428.

[bibr59-00031224241253268] Godoy Angelina Snodgrass . 2006. Popular Injustice: Violence, Community, and Law in Latin America. Stanford, CA: Stanford University Press.

[bibr60-00031224241253268] Goertz Gary . 2006. Social Science Concepts: A User’s Guide. Princeton, NJ: Princeton University Press.

[bibr61-00031224241253268] Goldstein Daniel M. 2003. “‘In Our Own Hands’: Lynching, Justice, and the Law in Bolivia.” American Ethnologist 30(1):22–43.

[bibr62-00031224241253268] Goldstein Daniel M. Achá Gloria Hinojosa Eric Roncken Theo . 2007. “La Mano Dura and the Violence of Civil Society in Bolivia.” Social Analysis 51(2):43–63.

[bibr63-00031224241253268] González Yanilda Mayka Lindsay . 2023. “Policing, Democratic Participation, and the Reproduction of Asymmetric Citizenship.” American Political Science Review 117(1):263–79.

[bibr64-00031224241253268] Granovetter Mark S. 1978. “Threshold Models of Collective Behavior.” American Journal of Sociology 83(6):1420–43.

[bibr65-00031224241253268] Granovetter Mark S. 1973. “The Strength of Weak Ties.” American Journal of Sociology 78(6):1360–80.

[bibr66-00031224241253268] Gutiérrez-Sanín Francisco Wood Elisabeth Jean . 2017. “What Should We Mean by ‘Pattern of Political Violence’? Repertoire, Targeting, Frequency, and Technique.” Perspectives on Politics 15(1):20–41.

[bibr67-00031224241253268] Hagen Ryan Makovi Kinga Bearman Peter . 2013. “The Influence of Political Dynamics on Southern Lynch Mob Formation and Lethality.” Social Forces 92(2):757–87.

[bibr68-00031224241253268] Handy Jim . 2004. “Chicken Thieves, Witches, and Judges: Vigilante Justice and Customary Law in Guatemala.” Journal of Latin American Studies 36(3):533–61.

[bibr69-00031224241253268] Haynie Dana L. 2001. “Delinquent Peers Revisited: Does Network Structure Matter?” American Journal of Sociology 106(4):1013–57.

[bibr70-00031224241253268] Herriman Nicholas . 2010. “The Great Rumor Mill: Gossip, Mass Media, and the Ninja Fear.” The Journal of Asian Studies 69(3):723–48.

[bibr71-00031224241253268] Horowitz Donald L. 2001. The Deadly Ethnic Riot. Berkeley: University of California Press.

[bibr72-00031224241253268] Infobae. 2020. “‘El coraje es el coraje’: la justicia por propia mano divide opiniones entre los mexicanos.” August 13.

[bibr73-00031224241253268] Instituto Belisario Domínguez. 2017. Recuento de Los Daños 7S y 19S: A Un Mes de La Tragedia. Senado de la República.

[bibr74-00031224241253268] Jackson Jonathan Huq Aziz Z. Bradford Ben Tyler Tom R. . 2013. “Monopolizing Force? Police Legitimacy and Public Attitudes toward the Acceptability of Violence.” Psychology, Public Policy, and Law 19(4):479–97.

[bibr75-00031224241253268] Jasper James M. 2011. “Emotions and Social Movements: Twenty Years of Theory and Research.” Annual Review of Sociology 37(1):285–303.

[bibr76-00031224241253268] Jones Bradford S. Norrander Barbara . 1996. “The Reliability of Aggregated Public Opinion Measures.” American Journal of Political Science 40(1):295–309.

[bibr77-00031224241253268] Jung Danielle F. Cohen Dara Kay . 2020. Lynching and Local Justice: Legitimacy and Accountability in Weak States. Cambridge, UK: Cambridge University Press.

[bibr78-00031224241253268] Kalyvas Stathis N. 2019. “The Landscape of Political Violence.” Pp. 11–33 in The Oxford Handbook of Terrorism. Oxford, UK: Oxford University Press.

[bibr79-00031224241253268] Karim Sabrina . 2020. “Relational State Building in Areas of Limited Statehood: Experimental Evidence on the Attitudes of the Police.” American Political Science Review 14(2):536–51.

[bibr80-00031224241253268] Kloppe-Santamaría Gema . 2020. In the Vortex of Violence: Lynching, Extralegal Justice, and the State in Post-Revolutionary Mexico. Berkeley: University of California Press.

[bibr81-00031224241253268] Kloppe-Santamaría Gema . 2021. “Deadly Rumors: Lynching, Hearsay, and Hierarchies of Credibility in Mexico.” Journal of Social History 55(1):85–104.

[bibr82-00031224241253268] Kovács Balázs Hsu Greta Sharkey Amanda . 2023. “The Stickiness of Category Labels: Audience Perception and Evaluation of Change in Creative Markets.” Management Science (10.1287/mnsc.2021.02070).

[bibr83-00031224241253268] Krakowski Krzysztof . 2021. “Adjustments to Gang Exposure in Early Adolescence.” Journal of Peace Research 59(3):337–52.

[bibr84-00031224241253268] Krakowski Krzysztof Kursani Shpend . 2023. “Why Do People Use Informal Justice? Experimental Evidence from Kosovo.” Journal of Experimental Political Science (10.1017/XPS.2023.18).

[bibr85-00031224241253268] Kreft Anne-Kathrin Agerberg Mattias . 2024. “Imperfect Victims? Civilian Men, Vulnerability, and Policy Preferences.” American Political Science Review 118(1):274–90.

[bibr86-00031224241253268] Lessing Benjamin . 2021. “Conceptualizing Criminal Governance.” Perspectives on Politics 19(3):854–73.

[bibr87-00031224241253268] Levi Margaret . 1996. “Social and Unsocial Capital: A Review Essay of Robert Putnam’s Making Democracy Work.” Politics & Society 24(1):45–55.

[bibr88-00031224241253268] Ley Sandra . 2022. “High-Risk Participation: Demanding Peace and Justice amid Criminal Violence.” Journal of Peace Research 59(6):794–809.

[bibr89-00031224241253268] Lichbach Mark Irving . 1995. The Rebel’s Dilemma. Ann Arbor: University of Michigan Press.

[bibr90-00031224241253268] Littman Rebecca Paluck Elizabeth Levy . 2015. “The Cycle of Violence: Understanding Individual Participation in Collective Violence.” Political Psychology 36(S1):79–99.

[bibr91-00031224241253268] Lope de Vega Félix . [1619] 1989. Fuenteovejuna. Madrid: Cátedra.

[bibr92-00031224241253268] Luhmann Niklas . 2000. Vertrauen: Ein Mechanismus Der Reduktion Sozialer Komplexität, 4th ed. Stuttgart, Germany: UTB.

[bibr93-00031224241253268] Lupu Noam Rodríguez Mariana . 2021. “Pulse of Democracy.” LAPOP, Vanderbilt University, Nashville, TN.

[bibr94-00031224241253268] Magaloni Beatriz Robles Gustavo Matanock Aila M. Diaz-Cayeros Alberto Romero Vidal . 2020. “Living in Fear: The Dynamics of Extortion in Mexico’s Drug War.” Comparative Political Studies 53(7):1124–74.

[bibr95-00031224241253268] Martinez Monica Muñoz . 2018. The Injustice Never Leaves You: Anti-Mexican Violence in Texas. Cambridge, MA: Harvard University Press.

[bibr96-00031224241253268] Martins José de Souza . 1991. “Lynchings – Life by a Thread: Street Justice in Brazil, 1979–1988.” Pp. 21–32 in Vigilantism and the State in Modern Latin America, edited by Huggins M. K. . New York: Greenwood.

[bibr97-00031224241253268] Marwell Gerald Oliver Pamela . 1993. The Critical Mass in Collective Action: A Micro-Social Theory. Cambridge, UK: Cambridge University Press.

[bibr98-00031224241253268] Mattiace Shannan Nonnenmacher Tomas . 2022. “Internal Migration to Yucatán, Mexico: Moving for Security.” Mexican Studies/Estudios Mexicanos 38(3):406–33.

[bibr99-00031224241253268] McDoom Omar Shahabudin . 2014. “Antisocial Capital: A Profile of Rwandan Genocide Perpetrators’ Social Networks.” Journal of Conflict Resolution 58(5):865–93.

[bibr100-00031224241253268] Mead George H. 1918. “The Psychology of Punitive Justice.” American Journal of Sociology 23(5):577–602.

[bibr101-00031224241253268] México Evalúa . 2023. Hallazgos 2022: Seguimiento y Evaluación de La Justicia Penal En México. Mexico City: México Evalúa.

[bibr102-00031224241253268] Moncada Eduardo . 2017. “Varieties of Vigilantism: Conceptual Discord, Meaning and Strategies.” Global Crime 18(4):403–23.

[bibr103-00031224241253268] Moncada Eduardo . 2023. “The Political Economy of Collective Vigilantism: Comparative Evidence from Mexico.” Comparative Politics 55(2):337–58.

[bibr104-00031224241253268] Montambeault Françoise Félix Annabelle Dias . 2021. “When Informality Matters: Participatory Security Reform and Mechanisms of Social Embeddedness in Nezahualcóyotl, Mexico.” Journal of Latin American Studies 53(3):465–92.

[bibr105-00031224241253268] Mukhopadhyay Dipali Howe Kimberly . 2023. Good Rebel Governance: Revolutionary Politics and Western Intervention in Syria. Cambridge, UK: Cambridge University Press.

[bibr106-00031224241253268] Nevels Cynthia Skove . 2007. Lynching to Belong: Claiming Whiteness through Racial Violence. Austin: Texas A&M University Press.

[bibr107-00031224241253268] Nivette Amy E. 2016. “Institutional Ineffectiveness, Illegitimacy, and Public Support for Vigilantism in Latin America.” Criminology 54(1):142–75.

[bibr108-00031224241253268] Nussio Enzo . 2023. “How Moral Beliefs Influence Collective Violence: Evidence from Lynching in Mexico.” Comparative Political Studies (10.1177/00104140231223747). PMC11573652 39568445

[bibr109-00031224241253268] Nussio Enzo . 2024. “Replication Data for: The ‘Dark Side’ of Community Ties: Collective Action and Lynching in Mexico” (10.7910/DVN/JOTLND). PMC11294007 39100989

[bibr110-00031224241253268] Nussio Enzo Clayton Govinda . 2023. “A Wave of Lynching: Morality and Authority in Post-Tsunami Aceh.” Comparative Politics 55(2):313–36.

[bibr111-00031224241253268] Nussio Enzo Clayton Govinda . 2024. “Introducing the Lynching in Latin America (LYLA) Dataset.” Journal of Peace Research (10.1177/00223433231220275).

[bibr112-00031224241253268] Nussio Enzo Oppenheim Ben . 2014. “Anti-Social Capital in Former Members of Non-state Armed Groups: A Case Study of Colombia.” Studies in Conflict & Terrorism 37(12):999–1023.

[bibr113-00031224241253268] Nussio Enzo Ugarriza Juan E. . 2021. “Why Rebels Stop Fighting: Organizational Decline and Desertion in Colombia’s Insurgency.” International Security 45(4):167–203.

[bibr114-00031224241253268] Obert Jonathan Mattiacci Eleonora . 2018. “Keeping Vigil: The Emergence of Vigilance Committees in Pre-Civil War America.” Perspectives on Politics 16(3):600–616.

[bibr115-00031224241253268] Olabuenaga Ana María . 2019. Linchamientos Digitales. Mexico City: Paidos.

[bibr116-00031224241253268] Olson Mancur . 1965. The Logic of Collective Action: Public Goods and the Theory of Groups. Cambridge, MA: Harvard University Press.

[bibr117-00031224241253268] Osorio Javier Schubiger Livia Isabella Weintraub Michael . 2021. “Legacies of Resistance: Mobilization against Organized Crime in Mexico.” Comparative Political Studies 54(9):1565–96.

[bibr118-00031224241253268] Oster Emily . 2019. “Unobservable Selection and Coefficient Stability: Theory and Evidence.” Journal of Business & Economic Statistics 37(2):187–204.

[bibr119-00031224241253268] Ostrom Elinor . 1990. Governing the Commons. Cambridge, UK: Cambridge University Press.

[bibr120-00031224241253268] Ostrom Elinor . 2000. “Social Capital: A Fad or a Fundamental Concept?” Pp. 172–214 in Social Capital: A Multifaceted Perspective, edited by Serageldin I. Dasgupta P. . Washington, DC: World Bank Publications.

[bibr121-00031224241253268] Pearl Judea Mackenzie Dana . 2018. The Book of Why: The New Science of Cause and Effect. London, UK: Hachette.

[bibr122-00031224241253268] Petersen Roger D. 2001. Resistance and Rebellion: Lessons from Eastern Europe. Cambridge, UK: Cambridge University Press.

[bibr123-00031224241253268] Pfeifer Michael J. 2004. Rough Justice: Lynching and American Society, 1874–1947. Chicago: University of Illinois Press.

[bibr124-00031224241253268] Piccato Pablo . 2017. A History of Infamy: Crime, Truth, and Justice in Mexico. Oakland: University of California Press.

[bibr125-00031224241253268] Portes Alejandro Landolt Patricia . 1996, May 1. “The Downside of Social Capital.” The American Prospect, pp. 18–21.

[bibr126-00031224241253268] Putnam Robert D. 1993. Making Democracy Work: Civic Traditions in Modern Italy. Princeton, NJ: Princeton University Press.

[bibr127-00031224241253268] Putnam Robert D. 2002. Democracies in Flux: The Evolution of Social Capital in Contemporary Society. Oxford, UK: Oxford University Press.

[bibr128-00031224241253268] Putzel James . 1997. “Accounting for the ‘Dark Side’ of Social Capital: Reading Robert Putnam on Democracy.” Journal of International Development 9(7):939–49.

[bibr129-00031224241253268] Rickard Kit Bakke Kristin M. . 2021. “Legacies of Wartime Order: Punishment Attacks and Social Control in Northern Ireland.” Security Studies 30(4):603–36.

[bibr130-00031224241253268] Robinson William S. 1950. “Ecological Correlations and the Behavior of Individuals.” American Sociological Review 15(3):351–57.

[bibr131-00031224241253268] Rodríguez Guillén Raúl Veloz Ávila Norma Ilse . 2019. “Linchamientos En México: Una Puesta al Día.” El Cotidiano 34(214):87–94.

[bibr132-00031224241253268] Sampson Robert J. 2008. “Collective Efficacy Theory: Lessons Learned and Directions for Future Inquiry.” Pp. 149–67 in Taking Stock: The Status of Criminological Theory, edited by Cullen F. T. Wright J. P. Blevins K. R. . London, UK: Routledge.

[bibr133-00031224241253268] Sampson Robert J. Raudenbush Stephen W. Earls Felton . 1997. “Neighborhoods and Violent Crime: A Multilevel Study of Collective Efficacy.” Science 277(5328):918–24. 10.1126/science.277.5328.918 9252316

[bibr134-00031224241253268] Satyanath Shanker Voigtländer Nico Voth Hans-Joachim . 2017. “Bowling for Fascism: Social Capital and the Rise of the Nazi Party.” Journal of Political Economy 125(2):478–526.

[bibr135-00031224241253268] Scacco Alexandra . 2012. Anatomy of a Riot: Participation in Ethnic Violence in Nigeria. Book manuscript, New York University.

[bibr136-00031224241253268] Scott James C. 1976. The Moral Economy of the Peasant: Rebellion and Subsistence in Southeast Asia. New Haven, CT: Yale University Press.

[bibr137-00031224241253268] Senechal de la Roche Roberta . 1996. “Collective Violence as Social Control.” Sociological Forum 11(1):97–128.

[bibr138-00031224241253268] Senechal de la Roche Roberta . 1997. “The Sociogenesis of Lynching.” Pp. 48–76 in Under Sentence of Death: Lynching in the South, edited by Brundage W. F. . Chapel Hill: University of North Carolina Press.

[bibr139-00031224241253268] Shadish William R. Cook Thomas D. Campbell Donald Thomas . 2002. Experimental and Quasi-Experimental Designs for Generalized Causal Inference. Boston: Houghton Mifflin.

[bibr140-00031224241253268] Sharkey Patrick Torrats-Espinosa Gerard Takyar Delaram . 2017. “Community and the Crime Decline: The Causal Effect of Local Nonprofits on Violent Crime.” American Sociological Review 82(6):1214–40.

[bibr141-00031224241253268] Skinner Chris J. 2016. “Probability Proportional to Size (PPS) Sampling.” Pp. 1–5 in Wiley StatsRef. New York: John Wiley & Sons.

[bibr142-00031224241253268] Smångs Mattias . 2016. “Doing Violence, Making Race: Southern Lynching and White Racial Group Formation.” American Journal of Sociology 121(5):1329–74. 10.1086/684438 27092388

[bibr143-00031224241253268] Smångs Mattias . 2017. Doing Violence, Making Race: Lynching and White Racial Group Formation in the U.S. South, 1882–1930. New York: Routledge.

[bibr144-00031224241253268] Smith Nicholas Rush . 2019. Contradictions of Democracy: Vigilantism and Rights in Post-Apartheid South Africa. Oxford, UK: Oxford University Press.

[bibr145-00031224241253268] Snow David A. Moss Dana M. . 2014. “Protest on the Fly: Toward a Theory of Spontaneity in the Dynamics of Protest and Social Movements.” American Sociological Review 79(6):1122–43.

[bibr146-00031224241253268] Solnit Rebecca . 2010. A Paradise Built in Hell: The Extraordinary Communities That Arise in Disaster. New York: Penguin.

[bibr147-00031224241253268] Tajima Yuhki . 2014. The Institutional Origins of Communal Violence: Indonesia’s Transition from Authoritarian Rule. Cambridge, UK: Cambridge University Press.

[bibr148-00031224241253268] Tankebe Justice . 2009. “Self-Help, Policing, and Procedural Justice: Ghanaian Vigilantism and the Rule of Law.” Law & Society Review 43(2):245–70.

[bibr149-00031224241253268] Taylor Michael . 1982. Community, Anarchy and Liberty. Cambridge, UK: Cambridge University Press.

[bibr150-00031224241253268] Theriault Jordan E. Young Liane Barrett Lisa Feldman . 2021. “The Sense of Should: A Biologically-Based Framework for Modeling Social Pressure.” Physics of Life Reviews 36:100–136. 32008953 10.1016/j.plrev.2020.01.004 PMC8645214

[bibr151-00031224241253268] Thompson Edward P. 1971. “The Moral Economy of the English Crowd in the Eighteenth Century.” Past & Present 50:76–136.

[bibr152-00031224241253268] Thurston Robert W. 2011. Lynching: American Mob Murder in Global Perspective. London, UK: Routledge.

[bibr153-00031224241253268] Tilly Charles . 1978. From Mobilization to Revolution. London, UK: McGraw-Hill.

[bibr154-00031224241253268] Tilly Charles . 2003. The Politics of Collective Violence. Cambridge, UK: Cambridge University Press.

[bibr155-00031224241253268] Tolnay Stewart E. Beck E. M. . 1995. A Festival of Violence: An Analysis of Southern Lynchings, 1882–1930. Urbana: University of Illinois Press.

[bibr156-00031224241253268] Trejo Guillermo Ley Sandra . 2020. Votes, Drugs, and Violence. Cambridge, UK: Cambridge University Press.

[bibr157-00031224241253268] Twain Mark . 1923. “The United States of Lyncherdom” (https://en.wikisource.org/wiki/The_United_States_of_Lyncherdom).

[bibr158-00031224241253268] UNODC. 2019. Global Study on Homicide. Vienna: UNODC.

[bibr159-00031224241253268] Varshney Ashutosh Staggs Connor . 2024. “Hindu Nationalism and the New Jim Crow.” Journal of Democracy 35(1):5–18.

[bibr160-00031224241253268] Vilalta Carlos J. Lopez Pablo Fondevila Gustavo Siordia Oscar . 2020. “Testing Broken Windows Theory in Mexico City.” Social Science Quarterly 101(2):558–72.

[bibr161-00031224241253268] Vilas Carlos M. 2001. “By Their Own Hands: Mass Lynching in Contemporary Mexico.” Southwestern Journal of Law and Trade in the Americas 8:311–34.

[bibr162-00031224241253268] Voswinkel Stephan . 2011. “Reputation: A Sociological View.” Pp. 31–45 in Reputation Management, Management for Professionals, edited by Helm S. Liehr-Gobbers K. Storck C. . Berlin: Springer.

[bibr163-00031224241253268] Waldrep Christopher . 2000. “War of Words: The Controversy over the Definition of Lynching, 1899–1940.” The Journal of Southern History 66(1):75–100.

[bibr164-00031224241253268] Weinstein Jeremy M. 2007. Inside Rebellion: The Politics of Insurgent Violence. Cambridge, UK: Cambridge University Press.

[bibr165-00031224241253268] Wells Ida B. 1892. Southern Horrors: Lynch Law in All Its Phases. New York.

[bibr166-00031224241253268] Welsh Bridget . 2008. “Local and National: Keroyokan Mobbing in Indonesia.” Journal of East Asian Studies 8(3):473–504.

[bibr167-00031224241253268] Westwood Sean J. Grimmer Justin Tyler Matthew Nall Clayton . 2022. “Current Research Overstates American Support for Political Violence.” Proceedings of the National Academy of Sciences 119(12):e2116870119 (10.1073/pnas.2116870119). PMC8944847 35302889

[bibr168-00031224241253268] Whitt Sam Wilson Rick K. . 2007. “Public Goods in the Field: Katrina Evacuees in Houston.” Southern Economic Journal 74(2):377–87.

[bibr169-00031224241253268] Wilke Anna M. 2022. “Gender Gaps in Support for Vigilante Violence.” Comparative Politics 55(2):263–85.

[bibr170-00031224241253268] Wilkinson Steven I. 2009. “Riots.” Annual Review of Political Science 12(1):329–43.

[bibr171-00031224241253268] Wood Elisabeth Jean . 2003. Insurgent Collective Action and Civil War in El Salvador. Cambridge, UK: Cambridge University Press.

[bibr172-00031224241253268] Wood George Tyler Tom R. Papachristos Andrew V. . 2020. “Procedural Justice Training Reduces Police Use of Force and Complaints against Officers.” Proceedings of the National Academy of Sciences 117(18):9815–21. 10.1073/pnas.1920671117 PMC7211954 32312803

[bibr173-00031224241253268] Wyatt-Brown Bertram . 1982. Southern Honor: Ethics and Behavior in the Old South. Oxford, UK: Oxford University Press.

[bibr174-00031224241253268] Yamamura Eiji . 2016. “Natural Disasters and Social Capital Formation: The Impact of the Great Hanshin-Awaji Earthquake.” Papers in Regional Science 95(S1):S143–64.

[bibr175-00031224241253268] Zizumbo-Colunga Daniel . 2015. “Taking the Law into Our Hands: Trust, Social Capital and Vigilante Justice.” PhD thesis, Vanderbilt University, Nashville, TN.

[bibr176-00031224241253268] Zizumbo-Colunga Daniel . 2017. “Community, Authorities, and Support for Vigilantism: Experimental Evidence.” Political Behavior 39(4):989–1015.

[bibr177-00031224241253268] Zizumbo-Colunga Daniel . 2019. “Confronting Crime by Ourselves: Trust in Neighbors, Trust in Authorities, and Anti-Crime Organization.” Latin American Research Review 54(3):574–90.

