# Supplementary material for: The “Dark Side” of Community Ties: Collective Action and Lynching in Mexico
Source: Am Sociol Rev. 2024 Jun 7;89(4):708–34. doi: 10.1177/00031224241253268 (PMC11294007; doi:10.1177/00031224241253268)
Supplement: sj-pdf-1-asr-10.1177_00031224241253268 – Supplemental material for The “Dark Side” of Community Ties: Collective Action and Lynching in Mexico [file sj-pdf-1-asr-10.1177_00031224241253268.pdf]

AMERICAN SOCIOLOGICAL REVIEW

OFFICIAL JOURNAL OF THE AMERICAN SOCIOLOGICAL ASSOCIATION

ONLINE SUPPLEMENT
to article in
AMERICAN SOCIOLOGICAL REVIEW, 2024, VOL. 89

The "Dark Side" of Community Ties: Collective Action and Lynching in Mexico

Enzo Nussio
ETH Zurich

Contents

A1. Mexico City survey
A2. Lynching data
A3. Mexico municipality data
A4. Additional analysis
A5. References

A1. Mexico City survey[1]

The survey questionnaire was prepared over the course of one and a half years, mainly in collaboration with the Mexican survey firm Data OPM and research assistants. The questionnaire drew in part on previously tested questions used in the Latin American Public Opinion Project surveys in Mexico, the largest survey program in Latin America.[2] Additional questions were developed independently in collaboration with the survey firm.

The vignette describing a typical lynching incident was inspired by a similar wording used in the survey of the Comisión Nacional de Derechos Humanos on lynching in Mexico (CNDH 2019). It is important to mention that the item about participation in lynching comes after the item about witnessing and standing by. Hence, it is clear from the question ordering that participation goes beyond mere bystanding.

Questions on community ties were piloted previously. The question on "people known by name in the neighborhood" was related to community participation and provided meaningful variation.

Piloting and questionnaire development

A previous version of the survey questionnaire was piloted twice on the Qualtrics Platform restricting the sample to residents of Mexico City. Once from June 25 to July 5, 2021 (*N* = 300). We do not refer to this pilot in the main text as the questionnaire changed drastically as a result. The second pilot was fielded from November 8 to 25, 2021 (*N* = 522). This pilot was also used to examine a list experiment for the participation in lynching question (García-Sánchez and Queirolo 2021).[3] Proportions from the list experiment and the direct

[1] Parts of the following description are published elsewhere as well.
[2] See https://www.vanderbilt.edu/lapop/
[3] The question of the list experiment was as follows: a randomly selected half of respondents received only three response options, and the other half also received option four: "Now, I am going to mention a series of [3/4] activities and I would like to know *how many* of them you have done in the past, not *which* ones. Simply add up the number of activities you have done, indicating a total number between 0 and [3/4]. At some point, have you (1) cleaned up a park in the neighborhood, (2) crossed the street when the traffic lights were red, (3) fixed the public lighting in your neighborhood [, (4) participated, with your neighbors, in the punishment of a thief]. How many of these activities have you ever done in the past? 0, 1, 2, 3 [, 4])"

question did not differ significantly, which is why we abstained from including the list experiment in the final questionnaire, to reduce its complexity.

The same vignette, including three key questions of the questionnaire (agreement with lynching, participation in lynching, and knowing about lynching in community), was fielded in November 2021 in a face-to-face Omnibus survey representative of the Mexican adult population (N = 1,019), resulting in very similar proportions as the ones recovered for the Mexico City survey.

Note on reliability of participation in lynching question

Readers may be concerned about the reliability of asking about participation in lynching. To examine this aspect, I did the following: First, I conducted two anonymous online pilot surveys. Online surveys are less susceptible to social desirability bias (Kreuter, Presser, and Tourangeau 2008). Using the Qualtrics platform[4] and restricting the sample selection to Mexico City (N = 522 and N = 300), 7.3 and 8 percent, respectively, answered that they had participated in a lynching-style incident, slightly below the 9.6 percent in our face-to-face sample.[5] Second, an identical vignette-based participation question was included in a survey representative of the adult population in the whole of Mexico in November 2021 (N = 1,019), conducted by the same survey firm as the Mexico City survey. In this survey, 10.3 percent of participants stated they had participated, close to our estimate for Mexico City. Given that the majority of respondents in the Mexico City survey agree with the perpetrators (71.3 percent), social desirability seems less problematic for our measurement.

Sampling

The survey targeted a high number of primary sampling units (colonias). The targeted number of 340 units was a compromise between sufficient dispersion across the city and a sufficiently large number of interviewees within each unit (at least six). The six individuals in each unit were block-randomized to the three treatment conditions (an element of the survey not used in this study[6]).

The 340 colonias were selected by the principal investigators using probability proportional to size (PPS) sampling (Skinner 2016) without replacement, implemented in Stata 16. There is no readily available information on colonia-level population. We therefore created a colonia-level indicator for population size based on census information for the street block, or "manzana," level (see Colonia-level dataset below) (Vilalta, Muggah, and Fondevila 2020). We also selected a secondary set of 70 replacement colonias using the same procedure, to allow for quick replacement in case the enumerators faced security problems or could not access buildings. In 19 colonias, interviews had to be complemented in directly adjacent areas or colonias had to be replaced completely (using colonias from the same *alcaldía* or borough). The main reason for this was restricted access to gated communities in wealthy neighborhoods. One colonia had to be replaced due to security concerns for the enumerators, a much lower number than initially expected.

The selection of households within colonias was identical to the procedures used by the widely tested Latin American Public Opinion Project. First, enumerators identified the Northeastern corner of a randomly chosen starting street block (*manzana*) within the selected colonia. From this point, the enumerators followed a random walk procedure, contacting every fifth household around the street block. Then, they selected a further street block using

[4] Online panels differ from the general population (Huff and Tingley 2015). Hence, these samples are exploratory and are not used for analysis.

[5] I also tested a list experiment in the second pilot (García-Sánchez and Queirolo 2021). It did not reveal a different proportion of affirmative answers than the direct question and thus was not used in the face-to-face survey.

[6] In additional analysis, I reran all analyses adjusting for these treatment conditions and results remain identical.

the same procedure until they reached the targeted six interviews reflecting an equal number of men and women, and an age distribution in line with census information. Given that a team of enumerators was working within the same colonia at the same time, certain colonias have slightly more than six interviews, but all have at least six interviews.

Survey fielding

The survey was implemented as a face-to-face survey due to the increased difficulty of reaching individuals by phone during the Covid-19 pandemic[7] and the limited possibilities in terms of geographically focused sampling in online surveys. Overall, the quality of a face-to-face survey was considered superior for our purposes.

A total of 61 previously trained enumerators conducted the survey between February 9 and March 1, 2022. The non-response rate was 44 percent. After introducing themselves, the enumerators began with a short description of the survey and handed out a consent sheet, including contact information for the study organizers and psychological counseling (see Ethical Considerations below). The average duration of the interview was 19 minutes. The last block of questions was directed to the enumerators, who filled them out without the presence of the respondents.

In the survey firm's field report, enumerators noted the following difficulties: encountering male participants as they are often out of the household and access to gated communities. Also, some questions had a difficult wording for participants with low levels of education. Advantages included the fact that enumerators could present themselves as working for a Swiss university and the topic of the survey (insecurity), which is of general interest to residents of Mexico City.

Survey monitoring

The survey implementation was monitored by 12 supervisors, three auditors, and two team coordinators, including in-person supervision of 15 percent of the sample and remote supervision of another 12 percent. The survey platform Survey To Go automatically recorded audio files of two short segments of the interview, without the enumerators' knowledge, to guarantee the effective application of the survey. Enumerators had to provide geographic and photographic information (not of the study participants) to show they effectively conducted the interview where they were supposed to conduct the interview and at the time they were supposed to conduct the interview. Interviews with evidence that enumerators did not conduct the survey appropriately were removed before starting the analysis. Unrealistically short interviews and interviews with overly fast progress from one question to the next were also removed. Identical responses to a series of consecutive questions were taken as a potential warning sign.

Links to questionnaire and consent sheet

Due to the length of the full survey questionnaire, I include a link to the original and translated versions.

Full survey questionnaire (original Spanish):
https://www.dropbox.com/s/fx9xdj2le5s9jnq/Questionnaire%20Espanol%20BLINDED.pdf?dl=0

Full survey questionnaire (English translation):

[7] According to Mexican survey firms, non-response rates of phone surveys increased considerably during the pandemic. In previous years, our survey partner needed roughly 25 calls for each completed survey; during the pandemic, they needed 130 calls for each completed survey.

https://www.dropbox.com/s/1j49hmkne1c5t5q/Questionnaire%20English%20BLINDED.pdf?dl=0

Consent sheet (original Spanish):
https://www.dropbox.com/s/d08z0m51h8bse4y/Consent%20sheet%20Espanol%20BLINDED.pdf?dl=0

Consent sheet (English translation):
https://www.dropbox.com/s/ttk9mc3jel0nh6n/Consent%20sheet%20English%20BLINDED.pdf?dl=0

Ethical considerations

In addition to the short references in the main text, here I report in more detail how I dealt with ethical challenges for the Mexico City survey, focusing mainly on risk mitigation strategies. The Ethics Board of ETH Zurich approved the proposed procedures.

This study did not create health risks for any participants, as we only collected information about their attitudes, beliefs, and narratives. Also, we do not foresee any societal-level or political risks stemming from this study. Similar research endeavors have been carried out in the past—for example, a survey on lynching in Mexico (CNDH 2019). Thus, the wider public is aware of the topic and direct political consequences are unlikely.

Our project is about collective violence. To better understand why collective violence occurs, we asked participants about their experience with and participation in collective violence. This is an important undertaking as it can potentially help us identify violence prevention tools, but it also has implications for ethically sound research. We are highly aware of potential risks and abstain from exposing participants to any excessive risks. We specifically focus here on psychological distress, legal implications, security risks for researchers, risks from the Corona pandemic, and data protection, and we discuss our strategies to mitigate these potential risks.

Psychological distress. After extended conversations with our local partners (Data OPM, legal counselor, and academic partners), we assessed the risk of re-traumatization during face-to-face interviews to be minimal. While the term lynching evokes grisly associations among a Western audience, who might think about brutal lynchings in the United States targeting African Americans in the nineteenth and early-twentieth centuries, Mexicans are constantly exposed to acts of self-justice, which usually amount to beatings of thieves, either in the news or through their own experience. This is also how we represent these events in our vignettes; we abstain from using the term lynching or “linchamiento.” For Mexicans, these events are normalized, as are surveys and studies about security-relevant questions. Hence, the risk of re-traumatization is minimal.

However, there may be rare cases in which study participants suffer emotional distress as a result of our surveys and interview questions. As a general stance, we avoid using value-laden language in our surveys and interviews. Also, we avoid talking about specific forms of violence that occurred during a lynching, as this is not the main focus of our inquiry. This should generally help limit the risk of psychological distress.

In preparing the surveys we made a further series of decisions to limit potential emotional distress. Our questions were asked in a non-intrusive way. Instead of asking directly about lynching, we presented respondents with a typical incident of a thief being punished by neighbors. The term lynching was avoided and other forms of extreme violence (e.g., burning, hanging) were not mentioned. Also, participants received contact details for free psychological counseling in case they felt uneasy after responding to our questions (see consent sheet below). These counseling options were independent from us, which local

partners indicated was preferable to connecting respondents with a specific service, which may be seen suspiciously.

Legal risks. Our survey asked about experiences with and participation in events similar to lynching. Identifying the drivers of lynching violence is a key contribution of our project. However, questions about experiences and participation are sensitive. In particular, the Mexican penal code requires citizens who have first-hand evidence of crimes to report them. This legal obligation did not affect our work, as we only received information about crimes via third-person referral.

We understand the tension between, on the one side, the moral obligation to report crimes when becoming aware of them and, on the other side, safeguarding the privacy of study participants. However, if this tension (reporting crimes versus privacy) was always solved in favor of the moral obligation to report crimes, many of the standard practices in studying violence would be impossible, including the very common practice of qualitative interviews in conflict contexts and victimization surveys, which are used around the globe. Given that a core goal of science is to contribute to the understanding and solution of important societal problems (such as violence), we argue that solving the tension in favor of protecting the privacy of study participants should be possible under appropriate conditions, to allow for research on the drivers and consequences of violence.

However, this has to be done responsibly, as described, for example, in the Manual on Victimization Surveys developed by the United Nations Office on Drugs and Crime (UNODC 2010). We devised a series of strategies to mitigate potential concerns. We developed these strategies in collaboration with the Mexican lawyer Alberto Abad Suárez Ávila, from the university UNAM, who has experience in public opinion surveys. In accordance with UNODC guidelines and local guidance from our Mexican partners, our strategy in collecting information about the experience and commission of violent acts was designed to avoid receiving any specific information on concrete crimes.

We did this by referring to hypothetical scenarios and asking general questions. With the information we received, it would not be possible to support the prosecution of crimes, unless we asked for additional information from our study participants, which was not in our interest. The information we did receive in no case determined a specific act of violence that could be reported to police or judicial institutions. In this sense, we avoided the tension between a moral obligation for reporting crimes and safeguarding respondents' privacy, as we are unable to report a concrete crime to authorities.

Specific approach taken with regard to the survey question "Have you ever participated in such an event? Yes, No, no answer." This question refers to a before-mentioned vignette: "A thief assaults a lady on the street. Using a knife, he takes her belongings and escapes. After the robbery, a passer-by manages to take away the thief's knife and subdues the thief. In this moment, a large number of people gather, insult and punish the thief."

We understand the sensitivity of our question, which is why we thought at length about how to formulate it. Before describing our strategy, the key here is that research on self-justice has exclusively focused on support for such violence but not participation in it. We believe that support and participation cannot be equalized, and that empirical research on this important societal problem would be largely improved if we had a more direct measure of participation.

Therefore, we developed a strategy (together with our Mexican legal counselor) to ask this question in a way that would not create a legal or moral obligation to contribute to prosecution. First, our participation question does not refer to an actual crime but to a hypothetical scenario. An affirmative answer—meaning the surveyee participated in a similar event—does not make it possible for us to contribute to the prosecution of a crime, as we are

not informed about any actual crime. We would have to ask additional questions to receive information on an actual crime that could be reported to authorities.

Second, the scenario does not necessarily describe a crime, as the outcome remains legally indeterminate. Citizens are described as insulting and punishing the thief, terms that have no clear legal implication.

Third, we do not specify the kind of participation. Hence, we do not know whether an affirmative answer to the participation question implies involvement of the surveyee in an actual crime or whether it describes active bystanding.

To sum up, given this framing of the question, it would be impossible for us to contribute to the prosecution of a crime.

In addition, no identifying information about the person who witnessed the hypothetical event or who participated in it was shared with us. Hence, we would not be able to report the participation of a given individual. Securing anonymity provides an additional layer of protection in our study set-up and has to be seen as part of a broader strategy to make it "impossible to draw any conclusions from the answers regarding the commitment of such crimes," as demanded by the Ethics Board.

Also, in line with the recommendation of our legal adviser, we included a note in the consent sheet and offered the possibility of psychological counseling (as mentioned above).

Safety risks for researchers and study participants. When studying violence, researchers may be exposed to risks emanating from their research sites. We devised a series of strategies to minimize risks for the researchers, in accordance with guidelines from our university's safety unit.

With regard to the surveys, we have long debated the mode of survey application. Phone and online survey modes have certain advantages when asking sensitive questions and are less costly, but face-to-face surveying has clear advantages in terms of sampling and data quality. The pilot survey was conducted online, but we decided to conduct the main survey face-to-face. This decision was the result of an extended conversation with our survey partners at DATA OPM. Given our questionnaire and sampling procedures, they estimated the risks for their enumerators to be minimal (in line with most other surveys they have done in the past). They also noted that phone survey participants became less responsive during the COVID-19 pandemic, making phone surveys in Mexico extremely burdensome.

The face-to-face survey had safety implications, especially for the enumerators, so our survey partner DATA OPM created a plan to ensure safety (Protocolo de Seguridad). For example, during the sampling procedure, locations known to be highly insecure were replaced. Furthermore, when enumerators encountered safety problems at a new location, they abandoned this location right away. This was not ideal from the perspective of random sampling, but we obviously weighed the safety of enumerators higher than minor methodological concerns.

Risks from the Covid-19 pandemic. The special situation of the Covid-19 pandemic necessitated additional safety measures. Researchers adhered to the guidelines of local authorities in Mexico and only traveled to field sites if the health risks of their study participants and themselves were manageable.

DATA OPM had their own additional guidelines to reduce risks for their enumerators ("Protocolo Sanitario por Contingencia COVID"); for example, no "high-risk" individuals were part of the enumerator team. Enumerators measured their temperature each day before starting the interviews, disinfected the screens of their mobile devices regularly, used face masks, maintained their distance from each other and the study participants, did not shake hands with participants, did not enter participants' households, and used private transportation rather than public transport.

The survey and field visits started no earlier than November 2021, as Mexico was on track to fully vaccinate their adult population by then.

Data protection. Data protection is particularly important in the context of our study, given that we accessed sensitive information. In principle, all information was anonymized before storage, safely stored on password protected computers, and was only accessible by the authors for the course of the study duration.

All data of the pilot online surveys (conducted with Qualtrics) were fully anonymized, hence, it was not possible for us to attribute any responses to specific individuals once we received the data from Qualtrics. This corresponds to Qualtrics' standard practice, which fully complies with EU's General Data Protection Regulation (GDPR).[8]

Data from the face-to-face survey (conducted by DATA OPM) were anonymized. Our survey partner followed the guidelines of the Mexican Federal Law of Personal Data Protection, and for this project, they also complied with EU's GDPR. Furthermore, their standard operating procedures comply with the ethical code of conduct and best practices of the most important public opinion research organizations (including the World Association for Public Opinion Research and the European Society for Opinion and Market Research). If DATA OPM receives information that allows for the identification of specific interviewees (e.g., the exact location of residence), this information is (a) not shared with the client, and (b) stored separately from the dataset that contains the survey responses. Specifically, DATA OPM creates an anonymized key for each respondent to which only their authorized employees have access. Secure file transfer services are used to transfer only the anonymized data at the end of the survey; the full information on respondents is stored and encrypted on DATA OPM's own server and erased after maximally six months.

Fully anonymized data of the survey (including questionnaires, codebooks, and replication code) will be made publicly available once the respective studies are published, as is standard practice in social science and following FAIR principles for scientific data management (Wilkinson et al. 2016). This is also in line with the spirit of our university's initiative on research data management. Publication of data facilitates replication of our analysis and thus prevents inappropriate data usage.

[8] See https://www.qualtrics.com/uk/platform/gdpr/

A2. Lynching data[9]

To identify and categorize lynching events, we primarily relied on Factiva, the most comprehensive global news database, containing almost two billion news articles from more than 33,000 news sources from 200 countries in around 28 languages (Nussio and Clayton 2024). This includes news networks, such as Reuters and the Associated Press, as well as local radio, television, and newspaper reporting in local languages.[10] Factiva allows researchers to search for specific keywords and specify the countries of interest.[11] We pilot tested several search strings, ultimately settling on a specification that included common terms for lynching in English and Spanish (e.g., lynching and linchamiento), a number of related colloquial terms in Spanish (e.g., *justicia por mano propia*), terms relating to mob violence in both Spanish and English (e.g., lynch mob and vigilantes), and excluding a number of common terms unrelated to our concept (e.g., the bank Merrill Lynch).

Next, we limited the geographic scope of our search to Latin America. Given that our data collection was mainly based on newspapers, we limited our focus to Spanish- and Portuguese-speaking countries in Latin America for reasons of language comparability. This includes Argentina, Bolivia, Brazil, Chile, Colombia, Costa Rica, Dominican Republic, Ecuador, El Salvador, Guatemala, Honduras, Mexico, Nicaragua, Panama, Paraguay, Peru, Uruguay, and Venezuela. We excluded Jamaica, Belize, Haiti, Guyana, Suriname, and a series of smaller Caribbean states. We also excluded Cuba due to limited newspaper reporting. The temporal focus for the whole of Latin America was 2010 to 2019 (for Mexico only, we collected data ranging from 2000 to February 2022). This approach produced a corpus of around 80,000 news articles.

Human coders then reviewed each article to determine whether it identified a lynching event. Identified cases of lynching were coded to capture key details such as the date of the event, the location, and number of targets and perpetrators,[12] as well as a series of descriptive variables, such as the form of violence, the alleged form of wrongdoing, and the physical consequences suffered.

Following best practices identified in prior projects collecting violent event data (Davenport and Moore 2015), all coding sheets were then checked by one of the more experienced coders. Any disagreements or contentious issues were either discussed in monthly coder meetings with the project leaders or solved between the coders bilaterally in the event of a clear error. Therefore, all lynching events included in the LYLA data were checked by at least two persons, and unclear cases reviewed by at least one project leader.

We set a low bar for events to enter the dataset, including cases that some may not consider lynchings but "attempted" lynchings. The boundary condition for inclusion was a clear threat of lynching violence. This allows researchers who use the dataset to set their own threshold, for example, including all LYLA cases, only cases resulting in injury, or, even more restrictively, only cases resulting in death.

The newspaper-based approach we adopt was the best suited to gather systematic cross-national lynching data, but this method has well-known limits when collecting violent event data. Reporting bias affects the collection of data on all types of violent events (Godoy 2006:26; Mendoza 2008:5; Weidmann 2015). News editors prioritize more newsworthy events, which means that more violent, urban, and spectacular lynchings involving unusual protagonists are reported more often (Miller et al. 2022; Odartey-Wellington and MacRae 2020). Our approach therefore risks introducing systematic bias (e.g., urban bias, bias toward

---

[9] I plan to use parts of the description used here in a separate article.
[10] We pilot tested LexisNexis and results were similar.
[11] We coded articles in English, Spanish, and Portuguese.
[12] A lynching usually involves three parties: a victim of an alleged wrongdoing (e.g., a victim of a theft), the alleged wrongdoer (e.g., a thief), and the perpetrators (the lynch mob). We refer to the alleged wrongdoer as the target rather than a victim to avoid confusion with the victim of the initially alleged wrongdoing.

bigger events). Similar problems also afflict well-known lynching datasets from the United States (Spilerman and Gerratana 2009). By relying on local media sources included within the Factiva database, we hope to mitigate some of these problems. Prior research has shown that despite these challenges this type of data can be instructive (Sundberg and Melander 2013).

On this note, while perhaps obvious to most readers, it is important to stress that the LYLA data capture *reported* lynchings. A significant number of lynchings go unreported in newspapers. Hence, we are not blind to potential biases in data collection and try to be as transparent as possible in our presentation. We also conducted additional validation checks using locally coded national datasets, which largely support the validity of our data. We provide a separate 50-page document including individual country reports comparing our data with other datasets.[13]

Importantly, our dataset recovers similar over-time variation and magnitudes of the phenomenon of lynching as other previously published datasets in Mexico. Figure A2.1 shows the development of reported lynchings in the LYLA dataset (black line) along with lynchings registered in other sources. We observe a generally increasing trend in the LYLA data. Another notable aspect of Figure 2.1 is the similarity in the pattern between the different sources. Between 2006 and 2011, Gamallo (2015) and Godínez (2017) have comparable observations. BBC Mundo's (2016) research collected 56 events in 2015, the CNDH (2019) report records 43 lynchings, and the LYLA dataset 98. Rodríguez and Veloz (2019) collected data for the period from 1988 to 2018. CEC records 150 lynching events for the year 2020.[14]

A2.1 Yearly evolution of lynching in different data sources

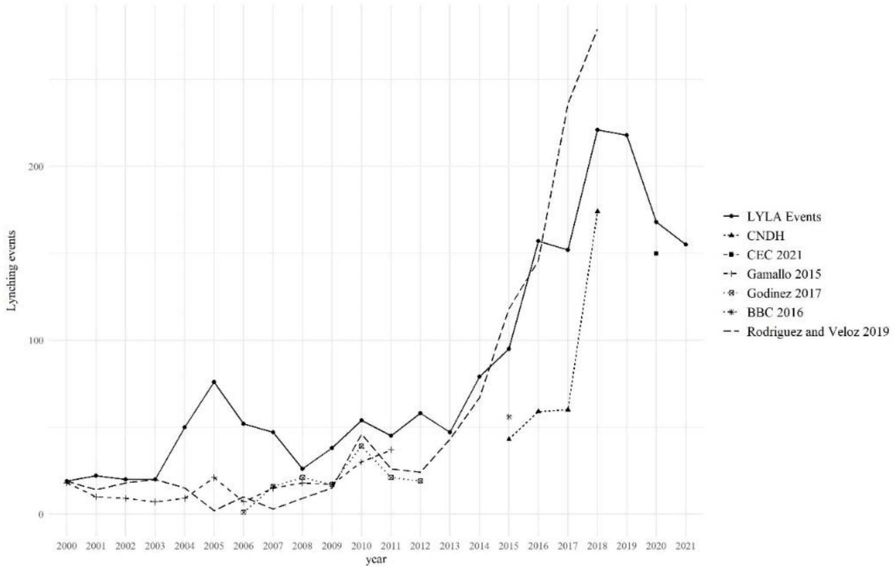

We opted to rely on newspaper reports after carefully considering alternatives, including crime statistics, social media, and surveys. First, lynching is not defined as a crime in the penal code of any Latin American state. A lynching incident may enter crime statistics as a homicide or injury, but given the large number of homicides and injuries unrelated to

[13] All supporting materials can be found here: https://css.ethz.ch/en/research/datasets/lynching-in-latin-america.html
[14] See http://causaencomun.org.mx/beta/wp-content/uploads/2021/01/210106_Informe-anual-atrocidades-2020_VF_compressed-1.pdf

lynchings, these forms of violence do not provide a meaningful proxy. Hence, there is no readily available official information on lynching.[15]

Second, we decided against using social media. News reports provide a relatively consistent corpus of data that can be analyzed systematically and retrospectively, and of which the biases are relatively well understood (Miller et al. 2022). Social media is harder to study systematically, and the biases are less clear and possibly quite different across contexts and time. Furthermore, social media entries on brutal violence tend to be quickly deleted from platforms. Using social media would also have risked exposing our coders to considerable psychological harm (Bellingcat 2018). We made sure our coders were only exposed to text, rather than to potentially more harmful visual material about lynching, shown on Facebook and other platforms.[16]

Third, we opted against using surveys for the systematic collection of lynching event data. To achieve sufficient coverage across time and space would have been prohibitively expensive, and would likely have revealed only scattered and geographically limited information about lynching events. Instead, we ran a survey to validate the LYLA data on the level of Mexico City neighborhoods. We also explored whether existing surveys might provide a workable source of data. However, we found possible indicators, such as expressed support for self-justice, to be relatively poor proxies for prevalence of lynching events (Agostini and van Zomeren 2021).[17]

---

[15] Some local administrations in Mexico recently started collecting information on lynching events, for example, the Secretaría General de Gobierno in Puebla State (Aguilar 2019).

[16] We prepared coders for the kind of content they were going to encounter before they started work, assured them they could stop working or take extended breaks without repercussions, and inquired about any disturbing experiences in monthly meetings, to provide a supportive environment.

[17] Pearson's correlation between average support for self-justice (taken from the Latin American Public Opinion Project) measured at the province level and lynching per million inhabitants is 0.09 (for other specifications of the lynching event variable, correlation is close to 0).

A3. Mexico municipality data

The municipality data used for independent variables and covariates (along with the lynching data) was pulled from different sources.

The most important source is the ENVIPE surveys (*Encuesta de Victimización y Percepción de Seguridad*) conducted by INEGI, the national statistics institute, a technocratic state institution producing high-quality data, including the national census.[18] We aggregated all ENVIPE surveys from 2011 to 2020 to generate mean municipality-level indicators. Given the large sample size of ENVIPE (N = 854,720), this provides good coverage of most municipalities in Mexico. Of the 2,457 Mexican municipalities, our indicators cover more than 1,500. The municipalities not covered are mostly micro-municipalities. For example, in Oaxaca alone, 399 extremely small municipalities are not covered for one of our key indicators (whether neighbors collaborated to deal with problems of public lighting). This is a result of the multi-stage random sampling procedure applied by the INEGI. The surveys were conducted usually between February and April each year. For each variable, I also collected how many observations were used to generate the municipality mean. This makes it possible to run the analysis on municipalities with particularly good coverage. I ran the main analyses with models including municipality means that are based on at least 10 survey respondents. In robustness checks, I ran the same analyses on a restricted sample of municipalities with at least 50 and 100 respondents, hence on a sample of generally larger municipalities, but with higher data quality. Results remained very similar.

For validation of the aggregate measures, I correlate a variable that exists in the ENVIPE surveys and in repeated national censuses. Pearson's correlation between the two measures is 0.87, suggesting the ENVIPE surveys are a valid reflection of municipality averages.

The analyses' main indicators based on ENVIPE are the following:

- Neighborly cooperation to deal with lighting: mean of asked people whose neighbors organized to deal with lighting problems
- Problem with lighting: mean of asked people who had problems with lack of lighting in their neighborhood
- Victimization: mean of asked people who suffered from a situation on the card [different types of crime] before the year before the survey
- Interpersonal trust: mean of asked people who trust neighbors
- Trust in army: mean of asked people who trust the army

Other variables used in the municipality analysis come from different state organizations:

- Population: INEGI[19]
- Surface area: derived from polygon data[20]
- Share of population living in poverty: Coneval[21]
- Gini coefficient: Coneval[22]
- Share of population speaking an indigenous language: INEGI[23]
- Share of non-religious population: INEGI[24]
- Homicide rate: INEGI[25] and SNSP[26] (cover different time periods)
- Robbery rate: SNSP[27]

---

[18] https://www.inegi.org.mx/programas/envipe/2022/
[19] https://en.www.inegi.org.mx/programas/ccpv/2000/
[20] http://conabio.gob.mx/informacion/metadata/gis/muni_2012gw.xml?&_xsl=/db/metadata/xsl/fgdc_html.xsl
[21] https://www.datos.gob.mx/busca/dataset/indicadores-de-pobreza-municipal-2010--2015
[22] https://www.coneval.org.mx/Medicion/Paginas/Cohesion_Social.aspx
[23] https://en.www.inegi.org.mx/programas/ccpv/2000/
[24] https://en.www.inegi.org.mx/programas/ccpv/2000/
[25] https://justiceinmexico.org/data/
[26] https://www.gob.mx/sesnsp/acciones-y-programas/datos-abiertos-de-incidencia-delictiva
[27] See https://www.gob.mx/sesnsp/acciones-y-programas/datos-abiertos-de-incidencia-delictiva

A4. Additional analysis

Individual-level analysis

A4.1 Full output for Table 2

	(1)	(2)	(3)	(4)
Names known (log)	.04***	.03***	.03***	.03***
	(.01)	(.01)	(.01)	(.01)
Trust	–.02*	–.02*	–.02*	–.02
	(.01)	(.01)	(.01)	(.01)
Education		.00	.00	.00
		(.00)	(.00)	(.00)
Age		–.00	.00	.00
		(.00)	(.00)	(.00)
Female		–.06***	–.02	–.02
		(.01)	(.01)	(.01)
Number of light bulbs		–.00	–.00	–.00
		(.00)	(.00)	(.00)
Unemployed		.01	.02	.02
		(.03)	(.03)	(.03)
Working			.00	.00
			(.01)	(.02)
Catholic			–.04*	–.06*
			(.02)	(.02)
Non-religious			–.04	–.06*
			(.02)	(.03)
Participated in fight			.14***	.14***
			(.02)	(.02)
Parents live in colonia			–.01	–.01
			(.02)	(.02)
Trust in government			–.00	–.00
			(.00)	(.01)
Garbage on the street			–.00	–.01
			(.01)	(.01)
Residential street block			–.02	–.01
			(.01)	(.02)
Constant	.04	.09*	.09	–.06
	(.03)	(.04)	(.05)	(.06)
N	2129	2117	2083	2083
Adj. R^2	.02	.03	.06	.08
Colonia FE	No	No	No	Yes
Control variables	No	Some	All	All

Note: Standard errors are in parentheses. OLS regression, standard errors clustered at colonia level (340 clusters). Varying *N* due to non-response.
*$p < 0.05$; **$p < 0.01$; ***$p < 0.001$. Two-tailed test.

A4.2 Community participation and lynching participation

Instead of names known in colonia, here I use the level of community participation to indicate community ties. The community participation indicator is the first component of participation in community meetings and participation in religious events.

	(1)	(2)	(3)	(4)
Community participation	.02*	.02**	.02**	.02**
	(.01)	(.01)	(.01)	(.01)
Constant	.10***	.14***	.10*	–.03
	(.01)	(.03)	(.05)	(.06)
N	2182	2169	2105	2105
Adj. R^2	.00	.01	.06	.07
Colonia FE	No	No	No	Yes
Control variables	No	Some	All	All

Note: Standard errors are in parentheses. OLS regression, standard errors clustered at colonia level (340 clusters). Varying *N* due to non-response.
**p* < 0.05; ***p* < 0.01; ****p* < 0.001. Two-tailed test.

A4.3 Community ties and lynching participation using logit modeling

	(1)	(2)	(3)	(4)
Names known (log)	.42***	.40***	.33***	.47***
	(.07)	(.07)	(.08)	(.11)
Trust	–.21*	–.23*	–.22*	–.28*
	(.09)	(.09)	(.09)	(.14)
Constant	–3.00***	–2.45***	–2.64***	–2.62**
	(.35)	(.46)	(.64)	(.97)
N	2129	2117	2060	947
Colonia FE	No	No	No	Yes
Control variables	No	Some	All	All

Note: Standard errors are in parentheses. OLS regression, standard errors clustered at colonia level (340 clusters). Varying *N* due to non-response.
**p* < 0.05; ***p* < 0.01; ****p* < 0.001. Two-tailed test.

A4.4 Community ties and lynching participation without outliers

Some respondents mention very high numbers of people known by name in their colonia. In the below analysis, I exclude outliers who say they know more than 200 people by name in their colonia.

	(1)	(2)	(3)	(4)
Names known (log)	.03***	.03***	.02***	.03***
	(.01)	(.01)	(.01)	(.01)
Trust	–.02*	–.02**	–.02*	–.02
	(.01)	(.01)	(.01)	(.01)
Constant	.06*	.11**	.12*	.00
	(.03)	(.04)	(.05)	(.06)
N	2099	2087	2030	2030
Adj. R^2	.02	.03	.06	.08
Colonia FE	No	No	No	Yes
Control variables	No	Some	All	All

Note: Standard errors are in parentheses. OLS regression, standard errors clustered at colonia level (340 clusters). Varying *N* due to non-response.
**p* < 0.05; ***p* < 0.01; ****p* < 0.001. Two-tailed test.

A4.5 Community ties and lynching participation, adjusting for witnessing a lynching

	(1)	(2)	(3)	(4)
Names known (log)	.03***	.02***	.02***	.03***
	(.01)	(.01)	(.01)	(.01)
Trust	–.01	–.01	–.01	–.01
	(.01)	(.01)	(.01)	(.01)
Witnessed a lynching	.22***	.22***	.20***	.19***
	(.02)	(.02)	(.02)	(.02)
Constant	–.00	.02	.02	–.12*
	(.03)	(.04)	(.05)	(.06)
N	2128	2116	2059	2059
Adj. R^2	.12	.12	.14	.14
Colonia FE	No	No	No	Yes
Control variables	No	Some	All	All

Note: Standard errors are in parentheses. OLS regression, standard errors clustered at colonia level (340 clusters). Varying *N* due to non-response.
*$p < 0.05$; **$p < 0.01$; ***$p < 0.001$. Two-tailed test.

A4.6 Community ties and lynching participation, restricted to individuals who witnessed a lynching (N = 498)

	(1)	(2)	(3)	(4)
Names known (log)	.05**	.05**	.05*	.09
	(.02)	(.02)	(.02)	(.05)
Trust	–.05*	–.05*	–.05*	–.01
	(.02)	(.02)	(.03)	(.06)
Constant	.23**	.24	.16	–.06
	(.09)	(.13)	(.17)	(.39)
N	489	488	478	478
Adj. R^2	.02	.01	.04	–.05
Colonia FE	No	No	No	Yes
Control variables	No	Some	All	All

Note: Standard errors are in parentheses. OLS regression, standard errors clustered at colonia level (340 clusters). Varying *N* due to non-response.
*$p < 0.05$; **$p < 0.01$; ***$p < 0.001$. Two-tailed test.

A4.7 Community ties and lynching participation, adjusting for agreeing with lynching

	(1)	(2)	(3)	(4)
Names known (log)	.03***	.03***	.03***	.03***
	(.01)	(.01)	(.01)	(.01)
Agrees with lynching	.05***	.05***	.05***	.05***
	(.01)	(.01)	(.01)	(.01)
Trust	–.02*	–.02*	–.02*	–.02
	(.01)	(.01)	(.01)	(.01)
Constant	–.11**	–.07	–.09	–.24***
	(.03)	(.04)	(.06)	(.07)
N	2103	2091	2037	2037
Adj. R^2	.04	.05	.08	.10
Colonia FE	No	No	No	Yes
Control variables	No	Some	All	All

Note: Standard errors are in parentheses. OLS regression, standard errors clustered at colonia level (340 clusters). Varying *N* due to non-response.
*$p < 0.05$; **$p < 0.01$; ***$p < 0.001$. Two-tailed test.

A4.8 Community ties and lynching participation, adjusting for previous lynchings in the colonia

	(1)	(2)	(3)	(4)
Names known (log)	.04***	.03***	.03***	.03***
	(.01)	(.01)	(.01)	(.01)
Trust	–.02*	–.02*	–.02*	–.02
	(.01)	(.01)	(.01)	(.01)
Lynching in colonia	.00	.00	.01	–.03
	(.01)	(.01)	(.01)	(.03)
Constant	.04	.09*	.08	–.07
	(.03)	(.04)	(.05)	(.06)
N	2129	2117	2060	2060
Adj. R^2	.02	.03	.07	.08
Colonia FE	No	No	No	Yes
Control variables	No	Some	All	All

Note: Standard errors are in parentheses. OLS regression, standard errors clustered at colonia level (340 clusters). Varying *N* due to non-response.
$*p < 0.05$; $**p < 0.01$; $***p < 0.001$. Two-tailed test.

A4.9 Parents' siblings and lynching participation

These models use parents' number of siblings as a temporally prior proxy for community ties.

	(1)	(2)	(3)	(4)
Parents' siblings in dozens	.05**	.06**	.04*	.04
	(.02)	(.02)	(.02)	(.02)
Constant	.05***	.10***	.07	–.07
	(.01)	(.03)	(.05)	(.06)
N	2182	2169	2105	2105
Adj. R^2	.00	.02	.05	.07
Colonia FE	No	No	No	Yes
Control variables	No	Some	All	All

Note: Standard errors are in parentheses. OLS regression, standard errors clustered at colonia level (340 clusters). Varying *N* due to non-response.
$*p < 0.05$; $**p < 0.01$; $***p < 0.001$. Two-tailed test.

Mechanisms

A4.10 Full output for Table 3

	(1) United with neighbors	(2) United with neighbors	(3) Neighbors encourage lynching	(4) Neighbors encourage lynching
Names known (log)	.20***	.20***	.13***	.10***
	(.02)	(.02)	(.02)	(.03)
Trust	.33***	.31***	–.05	–.03
	(.03)	(.03)	(.03)	(.04)
Education	–.03***	–.02*	–.01	–.01
	(.01)	(.01)	(.01)	(.01)
Age	.01***	.01***	–.01***	–.01**
	(.00)	(.00)	(.00)	(.00)
Female	.00	–.05	–.33***	–.25***
	(.04)	(.05)	(.05)	(.07)
Number of light bulbs	–.00	–.00	.00	.01
	(.00)	(.00)	(.00)	(.01)
Own siblings		–.00		.02
		(.01)		(.01)
Catholic		–.02		–.06
		(.07)		(.09)
Non-religious		–.16*		–.17
		(.08)		(.10)
Working		–.02		.05
		(.05)		(.07)
Unemployed		–.17		.03
		(.11)		(.13)
Garbage on the street		–.01		.01
		(.04)		(.05)
Residential street block		.09		.02
		(.06)		(.07)
Participated in fight		–.10		.27***
		(.06)		(.07)
Parents live in colonia		.08		.10
		(.05)		(.06)
Trust in government		.07***		–.05*
		(.02)		(.02)
Constant	.97***	.79***	2.43***	2.14***
	(.10)	(.19)	(.14)	(.25)
N	2114	2057	2047	1993
Adj. R^2	.17	.19	.05	.09
Colonia FE	No	Yes	No	Yes
Control variables	Some	All	Some	All

Note: Standard errors are in parentheses. OLS regression, standard errors clustered at colonia level (340 clusters). Varying *N* due to non-response.
$*p < 0.05$; $**p < 0.01$; $***p < 0.001$. Two-tailed test.

A4.11 Alternative mechanisms

Here, I examine whether community ties and trust are related to alternative mechanisms that may explain participation in lynching. I focus on whether individuals feel threatened by delinquency (1 to 4 Likert scale), which should increase participation in lynching, have trust in government (1 to 7 Likert scale), which should decrease participation in lynching, and binding or collectivist values, measured with the moral foundations questionnaire (Graham et al. 2011), which should increase participation in lynching. Community ties are not systematically related to any of these alternative mechanisms. Interpersonal trust is, as expected, positively related to trust in government. In summary, none of these alternative mechanisms can account for the relationship between community ties and lynching

participation.

Different from the models reported in the main paper, Model 4 does not adjust for trust in government, as trust in government is the dependent variable.

	(1) Threatened by delinquency	(2) Threatened by delinquency	(3) Trust in government	(4) Trust in government	(5) Binding values	(6) Binding values
Names known	.00	.00	–.07*	–.04	.01	–.01
(log)	(.02)	(.02)	(.03)	(.04)	(.01)	(.02)
Trust	–.03	–.03	.26***	.25***	.00	–.03
	(.02)	(.03)	(.04)	(.05)	(.02)	(.02)
Constant	3.37***	3.19***	3.70***	4.21***	3.21***	3.53***
	(.11)	(.19)	(.19)	(.29)	(.09)	(.16)
N	2112	2056	2084	2060	2118	2060
Adj. R^2	.05	.08	.02	.10	.04	.14
Colonia FE	No	Yes	No	Yes	No	Yes
Control variables	Some	All	Some	All	Some	All

Note: Standard errors are in parentheses. OLS regression, standard errors clustered at colonia level (340 clusters). Varying *N* due to non-response.
*p < 0.05; **p < 0.01; ***p < 0.001. Two-tailed test.

Aggregate-level analysis

A4.12 Full output for Table 4

	(1)	(2)	(3)	(4)
Neighborly cooperation	2.13**	2.54***	2.18***	1.31***
	(.60)	(.57)	(.58)	(.38)
Trust	–1.90*	–1.95**	–1.01	–.27
	(.73)	(.68)	(.51)	(.39)
Problem with lighting	.19	.58	.45	–.27
	(.48)	(.49)	(.43)	(.37)
Population		.00***	.00**	.00***
		(.00)	(.00)	(.00)
Area in km^2		–.00	–.00	.00
		(.00)	(.00)	(.00)
Poverty rate		–.40	.65	.15
		(.58)	(.61)	(.33)
Gini coefficient		–1.06	–1.78	1.20
		(1.70)	(1.54)	(1.30)
Share indigenous people		–.86	–.61	–.17
		(.52)	(.49)	(.20)
Non-religious population		2.17	1.80	2.17**
		(1.32)	(1.13)	(.83)
Homicide rate		–.00	–.01*	–.00
		(.00)	(.00)	(.00)
Robbery rate		–.08	.01	.03
		(.05)	(.04)	(.11)
Household victimization			4.44***	2.86***
			(.76)	(.50)
Trust in army			–1.32	–.86
			(.89)	(.63)
Constant	1.00	1.32	.64	–.68
	(.57)	(.85)	(1.05)	(.95)
N	1589	1589	1557	1557
Adj. R^2	.04	.11	.17	.27
Control variables	No	Some	All	All
Estado FE	No	No	No	Yes
Estado Clustered SE	Yes	Yes	Yes	No

Note: Standard errors are in parentheses.
$*p < 0.05$; $**p < 0.01$; $***p < 0.001$. Two-tailed test.

A4.13 Community ties, trust, and homicide rate (log)

	(1)	(2)	(3)	(4)
Neighborly cooperation	–1.06*	–.73	–.92*	–.25
	(.47)	(.39)	(.37)	(.18)
Trust	.45	.09	.10	–.29
	(.29)	(.35)	(.34)	(.18)
Constant	3.02***	.57	.09	–.07
	(.31)	(1.30)	(1.15)	(.44)
N	1589	1589	1557	1557
Adj. R^2	.03	.16	.16	.50
Control variables	No	Some	All	All
Estado FE	No	No	No	Yes
Estado clustered SE	Yes	Yes	Yes	No

Note: Standard errors are in parentheses.
$*p < 0.05$; $**p < 0.01$; $***p < 0.001$. Two-tailed test.

A4.14 Community ties, trust, and robbery rate (log)

	(1)	(2)	(3)	(4)
Neighborly cooperation	–.26	–.14	–.12	.00
	(.24)	(.15)	(.13)	(.04)
Trust	.07	.00	–.13	–.03
	(.06)	(.07)	(.16)	(.04)
Constant	.18	.63	.38	–.13
	(.15)	(.56)	(.34)	(.09)
N	1589	1589	1557	1557
Adj. R^2	.02	.08	.10	.72
Control variables	No	Some	All	All
Estado FE	No	No	No	Yes
Estado clustered SE	Yes	Yes	Yes	No

Note: Standard errors are in parentheses.
**p* < 0.05; ***p* < 0.01; ****p* < 0.001. Two-tailed test.

A4.15 Community ties, trust, and lynching rate (log)

In these models, I use the first principal component of three items of neighborly cooperation dealing with lighting, water, and water leakage problems. This procedure reduces the number of covered municipalities, but results remain consistent.

The three items used for principal component analysis have scale reliability alpha: 0.85

	(1)	(2)	(3)	(4)
Neighborly cooperation (principal component)	.12*	.22***	.23***	.11**
	(.05)	(.05)	(.06)	(.04)
Trust	–1.77*	–1.88*	–.80	.27
	(.80)	(.78)	(.62)	(.46)
Constant	2.09***	3.28*	2.29	.22
	(.54)	(1.23)	(1.30)	(1.03)
N	1396	1396	1387	1387
Adj. R^2	.04	.12	.18	.29
Control variables	No	Some	All	All
Estado FE	No	No	No	Yes
Estado clustered SE	Yes	Yes	Yes	No

Note: Standard errors are in parentheses.
**p* < 0.05; ***p* < 0.01; ****p* < 0.001. Two-tailed test.

A4.16 Community ties, trust, and lynching rate (not logged) using Poisson regression

	(1)	(2)	(3)	(4)
Neighborly cooperation	2.94***	2.49*	2.39*	1.18***
	(.88)	(.97)	(1.09)	(.06)
Trust	–1.79*	–1.83**	–1.14*	–.35***
	(.73)	(.60)	(.49)	(.06)
Constant	2.51*	5.09***	5.11*	.86***
	(1.07)	(1.31)	(2.54)	(.23)
N	1589	1589	1557	1557
Adj. R^2				
Control variables	No	Some	All	All
Estado FE	No	No	No	Yes
Estado clustered SE	Yes	Yes	Yes	No

Note: Standard errors are in parentheses. Poisson regression with cross-sectional municipality data and different specifications.
**p* < 0.05; ***p* < 0.01; ****p* < 0.001. Two-tailed test.

A4.17 Community ties, trust, and logged rate of lynchings with reported fatality or injury

	(1)	(2)	(3)	(4)
Neighborly cooperation	1.32**	1.64***	1.43***	1.04***
	(.38)	(.36)	(.35)	(.32)
Trust	–1.13	–1.06	–.43	.00
	(.55)	(.54)	(.45)	(.32)
Constant	.66	.45	.17	–.31
	(.42)	(.64)	(.79)	(.78)
N	1589	1589	1557	1557
Adj. R^2	.02	.09	.12	.20
Control variables	No	Some	All	All
Estado FE	No	No	No	Yes
Estado clustered SE	Yes	Yes	Yes	No

Note: Standard errors are in parentheses.
*$p < 0.05$; **$p < 0.01$; ***$p < 0.001$. Two-tailed test.

A4.18 Community ties, trust, and fatal lynching rate (log)

	(1)	(2)	(3)	(4)
Neighborly cooperation	.43**	.43**	.33*	.19
	(.15)	(.15)	(.15)	(.21)
Trust	–.24	–.13	.10	.22
	(.29)	(.32)	(.28)	(.21)
Constant	.15	–.13	.23	.10
	(.23)	(.39)	(.92)	(.52)
N	1589	1589	1557	1557
Adj. R^2	.00	.02	.03	.07
Control variables	No	Some	All	All
Estado FE	No	No	No	Yes
Estado clustered SE	Yes	Yes	Yes	No

Note: Standard errors are in parentheses. Linear regression with cross-sectional municipality data and different specifications.
*$p < 0.05$; **$p < 0.01$; ***$p < 0.001$. Two-tailed test.

A4.19 Community ties, trust, and lynching rate (log) – limited to municipalities with at least 50 ENVIPE individual observations for each municipality

	(1)	(2)	(3)	(4)
Neighborly cooperation	2.51*	3.63**	2.88**	1.42*
	(1.11)	(1.03)	(.98)	(.59)
Trust	–2.44*	–2.22	–.31	.89
	(1.15)	(1.11)	(.84)	(.62)
Constant	1.31	1.62	.26	–1.04
	(.82)	(1.20)	(1.28)	(1.24)
N	1050	1050	1049	1049
Adj. R^2	.05	.12	.19	.34
Control variables	No	Some	All	All
Estado FE	No	No	No	Yes
Estado clustered SE	Yes	Yes	Yes	No

Note: Standard errors are in parentheses. Linear regression with cross-sectional municipality data and different specifications.
*$p < 0.05$; **$p < 0.01$; ***$p < 0.001$. Two-tailed test.

A4.20 Community ties, trust, and lynching rate (log) – limited to municipalities with at least 100 ENVIPE individual observations for each municipality

	(1)	(2)	(3)	(4)
Neighborly cooperation	2.68*	4.11***	3.40**	1.33
	(1.12)	(1.02)	(1.03)	(.82)
Trust	–5.01**	–4.15**	–2.09	–.40
	(1.51)	(1.47)	(1.37)	(.93)
Constant	3.47***	3.33*	2.20	.85
	(.89)	(1.28)	(1.84)	(1.72)
N	690	690	690	690
Adj. R^2	.07	.15	.20	.33
Control variables	No	Some	All	All
Estado FE	No	No	No	Yes
Estado clustered SE	Yes	Yes	Yes	No

Note: Standard errors are in parentheses. Linear regression with cross-sectional municipality data and different specifications. The number of municipality-level observations is smaller than in other models, because I only include municipalities with a larger sample of ENVIPE individuals.
*$p < 0.05$; **$p < 0.01$; ***$p < 0.001$. Two-tailed test.

A4.21 Community ties, trust, and lynching rate (log) – limited to municipalities with at least 300 ENVIPE individual observations for each municipality

	(1)	(2)	(3)	(4)
Neighborly cooperation	3.48*	5.48***	4.53**	3.74*
	(1.43)	(1.38)	(1.55)	(1.52)
Trust	–7.38**	–5.11*	–3.11	–2.25
	(2.53)	(2.30)	(2.47)	(1.80)
Constant	5.13**	2.97	2.85	.14
	(1.42)	(1.83)	(2.38)	(3.05)
N	248	248	248	248
Adj. R^2	.14	.26	.29	.46
Control variables	No	Some	All	All
Estado FE	No	No	No	Yes
Estado clustered SE	Yes	Yes	Yes	No

Note: The number of municipality-level observations is smaller than in other models, because I only include municipalities with a larger sample of ENVIPE individuals. Standard errors are in parentheses. Linear regression with cross-sectional municipality data and different specifications.
*$p < 0.05$; **$p < 0.01$; ***$p < 0.001$. Two-tailed test.

Natural experiment

A4.22 Full output for Table 5

	(1)	(2)	(3)	(4)	(5)	(6)
Within 250km from earthquake × After 2017	.11***	.11***				
	(.02)	(.02)				
Earthquake damage × After 2017			.09***	.13***		
			(.02)	(.03)		
Distance from earthquake in 100km × After 2017					–.01***	–.01***
					(.00)	(.00)
2000.year	.00		.00		.00	
	(.)		(.)		(.)	
2001.year	.00		.00		.00	
	(.00)		(.00)		(.00)	
2002.year	.00		.00		.00	
	(.00)		(.00)		(.00)	
2003.year	.00		.00		.00	
	(.00)		(.00)		(.00)	
2004.year	.01***		.01***		.01***	
	(.00)		(.00)		(.00)	
2005.year	.02***		.02***		.02***	
	(.00)		(.00)		(.00)	
2006.year	.01**		.01**		.01**	
	(.00)		(.00)		(.00)	
2007.year	.01**		.01**		.01**	
	(.00)		(.00)		(.00)	
2008.year	.00		.00		.00	
	(.00)		(.00)		(.00)	
2009.year	.01*		.01*		.01*	
	(.00)		(.00)		(.00)	
2010.year	.01**		.01**		.01**	
	(.00)		(.00)		(.00)	
2011.year	.01**	.00	.01**	.00	.01**	.00
	(.00)	(.)	(.00)	(.)	(.00)	(.)
2012.year	.02***	.01	.02***	.01	.02***	.01
	(.00)	(.01)	(.00)	(.01)	(.00)	(.01)
2013.year	.01**	–.00	.01**	–.00	.01**	–.00
	(.00)	(.01)	(.00)	(.01)	(.00)	(.01)
2014.year	.03***	.03***	.03***	.03***	.03***	.03***
	(.01)	(.01)	(.01)	(.01)	(.01)	(.01)
2015.year	.03***	.04***	.03***	.04***	.03***	.05***
	(.01)	(.01)	(.01)	(.01)	(.01)	(.01)
2016.year	.06***	.07***	.06***	.07***	.06***	.07***
	(.01)	(.01)	(.01)	(.01)	(.01)	(.01)
2017.year	.05***	.07***	.05***	.08***	.05***	.07***
	(.01)	(.01)	(.01)	(.01)	(.01)	(.01)
2018.year	.04***	.05***	.05***	.05***	.13***	.14***
	(.01)	(.01)	(.01)	(.01)	(.02)	(.02)
2019.year	.04***	.06***	.04***	.06***	.12***	.15***
	(.01)	(.01)	(.01)	(.01)	(.02)	(.02)
2020.year	.02**		.02***		.10***	
	(.01)		(.01)		(.02)	
Homicides		.00		.00		.00
		(.00)		(.00)		(.00)
Robberies		.00		.00		.00
		(.00)		(.00)		(.00)
Kidnappings		.01		.01		.01
		(.01)		(.01)		(.01)
Infant mortality		–.01**		–.01**		–.01**

		(.00)		(.00)		(.00)
Constant	.01*	.07*	.01*	.07*	.01*	.07*
	(.00)	(.03)	(.00)	(.03)	(.00)	(.03)
N	51597	17225	51597	17225	51597	17225
Adj. R^2	.02	.07	.02	.07	.01	.06
Control variables	No	Yes	No	Yes	No	Yes

Note: Standard errors are in parentheses. Linear regression with year and municipality fixed effects, and municipality clustered standard errors.
*p < 0.05; **p < 0.01; ***p < 0.001. Two-tailed test.

NOTE: For this analysis, I do not have a reliable measure of time-varying community ties. Using the same ENVIPE survey wave data as for the cross-sectional analysis above, most individual municipality-year observations would be based on a very small sample of survey respondents and the analysis would thus be based on high levels of random measurement error. For comparison, the median number of survey respondents per municipality-year is 14 and the mean 38, whereas the median of survey respondents per municipality used in the cross-sectional analysis above is 61 and the mean 217. This allows me to restrict analysis to only those municipalities with precise measures (50, 100, and 300 respondents per municipality – see A4.16) but would create an unbalanced and highly restricted sample for municipality-year analysis.

A4.23 Earthquake exposure and newspaper reporting

An increase in lynching reports may be due to additional attention to areas exposed by the earthquake. Therefore, I compare the total news reporting in areas exposed and not exposed to the earthquake. The below reported dependent variable for this analysis is the number of news reports in the 40 largest municipalities of Mexico in each year. Collection of this information was restricted to the 40 largest municipalities of the country, as subnational news searches based on smaller municipalities would have generated too much noise. This explains the lower *N* for the analysis. The 40 municipalities are spread across the geography, including areas exposed and not exposed to the earthquake. If anything, news reporting decreases in earthquake-exposed municipalities, although the coefficients are not significant. In any case, increased lynchings are not a result of increased reporting.

	(1)	(2)	(3)	(4)	(5)	(6)
Within 250km from	–2631.35	–1968.87				
earthquake × After 2017	(4584.94)	(4134.60)				
Earthquake damage ×			–2631.35	–1968.87		
After 2017			(4584.94)	(4134.60)		
Distance from earthquake					243.43	208.48
in 100km × After 2017					(306.35)	(399.14)
Constant	862.17	10845.56***	862.17	10845.56***	862.17	10283.78**
	(999.97)	(2965.20)	(999.97)	(2965.20)	(1000.56)	(3514.94)
N	840	360	840	360	840	360
Adj. R^2	.41	.39	.41	.39	.41	.39
Control variables	No	Yes	No	Yes	No	Yes

Note: Standard errors are in parentheses. Linear regression with year and municipality fixed effects, and municipality clustered standard errors.
*p < 0.05; **p < 0.01; ***p < 0.001. Two-tailed test.

A4.24 Earthquake exposure and lynchings, adjusting for newspaper reporting

In this analysis, I adjust for the number of news reports per municipality. This analysis is restricted to the 40 largest municipalities. Results point in the same direction as in the main text, despite the limited number of observations.

	(1)	(2)	(3)	(4)	(5)	(6)
Within 250km from earthquake × After 2017	3.26***	3.17***				
	(.83)	(.88)				
Earthquake damage × After 2017			3.26***	3.17***		
			(.83)	(.88)		
Distance from earthquake in 100km × After 2017					–.19**	–.19**
					(.07)	(.07)
Number of news reports	.00**	.00	.00**	.00	.00*	.00
	(.00)	(.00)	(.00)	(.00)	(.00)	(.00)
Constant	.05	.06	.05	.06	.05	.99
	(.12)	(.74)	(.12)	(.74)	(.13)	(.75)
N	840	360	840	360	840	360
Adj. R^2	.36	.42	.36	.42	.27	.32
Control variables	No	Yes	No	Yes	No	Yes

Note; Standard errors are in parentheses. Linear regression with year and municipality fixed effects, and municipality clustered standard errors.
*$p < 0.05$; **$p < 0.01$; ***$p < 0.001$. Two-tailed test.

A4.25 Earthquake exposure and lynching, adjusting for lagged dependent variable

Use of a lagged dependent variable in two-way fixed-effects models is debated (Angrist and Pischke 2008). However, results remain consistent.

	(1)	(2)	(3)	(4)	(5)	(6)
Within 250km from earthquake × After 2017	.07***	.09***				
	(.01)	(.01)				
Earthquake damage × After 2017			.06***	.10***		
			(.01)	(.02)		
Distance from earthquake in 100km × After 2017					–.01***	–.01***
					(.00)	(.00)
Lagged DV	.34***	.28**	.34***	.28**	.34***	.28**
	(.08)	(.09)	(.08)	(.09)	(.08)	(.09)
Constant	–.01	.06*	–.01*	.05*	–.01*	.06*
	(.01)	(.03)	(.01)	(.03)	(.01)	(.03)
N	51596	17225	51596	17225	51596	17225
Adj. R^2	.13	.12	.13	.12	.13	.12
Control variables	No	Yes	No	Yes	No	Yes

Note: Standard errors are in parentheses. Linear regression with year and municipality fixed effects, and municipality clustered standard errors.
*$p < 0.05$; **$p < 0.01$; ***$p < 0.001$. Two-tailed test.

A4.26 Earthquake exposure and lynching, without year 2017

	(1)	(2)	(3)	(4)	(5)	(6)
Within 250km from earthquake × After 2017	.11***	.14***				
	(.02)	(.03)				
Earthquake damage × After 2017			.09***	.18***		
			(.02)	(.04)		
Distance from earthquake in 100km × After 2017					–.01***	–.01***
					(.00)	(.00)
Constant	.01*	.09*	.01*	.08*	.01*	.09*
	(.00)	(.04)	(.00)	(.04)	(.00)	(.04)
N	49140	14775	49140	14775	49140	14775
Adj. R^2	.02	.08	.02	.08	.01	.07
Control variables	No	Yes	No	Yes	No	Yes

Note: Standard errors are in parentheses. Linear regression with year and municipality fixed effects, and municipality clustered standard errors.
**p* < 0.05; ***p* < 0.01; ****p* < 0.001. Two-tailed test.

A4.27 Earthquake exposure and lynching, 2016 to 2018

	(1)	(2)	(3)	(4)	(5)	(6)
Within 250km from earthquake × After 2017	.07***	.06***				
	(.02)	(.02)				
Earthquake damage × After 2017			.06**	.06**		
			(.02)	(.02)		
Distance from earthquake in 100km × After 2017					–.01*	–.00*
					(.00)	(.00)
Constant	.06***	.01	.06***	.01	.06***	.01
	(.00)	(.05)	(.00)	(.05)	(.00)	(.05)
N	7371	6820	7371	6820	7371	6820
Adj. R^2	.01	.06	.01	.06	.00	.06
Control variables	No	Yes	No	Yes	No	Yes

Note: Standard errors are in parentheses. Linear regression with year and municipality fixed effects, and municipality clustered standard errors.
**p* < 0.05; ***p* < 0.01; ****p* < 0.001. Two-tailed test.

A4.28 Earthquake exposure and fatal lynchings

	(1)	(2)	(3)	(4)	(5)	(6)
Within 250km from earthquake × After 2017	.01***	.02***				
	(.00)	(.01)				
Earthquake damage × After 2017			.01***	.02***		
			(.00)	(.01)		
Distance from earthquake in 100km × After 2017					–.00***	–.00***
					(.00)	(.00)
Constant	.00*	.02*	.00*	.02*	.00*	.02*
	(.00)	(.01)	(.00)	(.01)	(.00)	(.01)
N	51597	17225	51597	17225	51597	17225
Adj. R^2	.00	.02	.00	.02	.00	.02
Control variables	No	Yes	No	Yes	No	Yes

Note: Standard errors are in parentheses. Linear regression with year and municipality fixed effects, and municipality clustered standard errors.
**p* < 0.05; ***p* < 0.01; ****p* < 0.001. Two-tailed test.

A4.29 Earthquake exposure and lynching, using Poisson analysis

	(1)	(2)	(3)	(4)	(5)	(6)
Within 250km from earthquake × After 2017	.62***	.64***				
	(.14)	(.19)				
Earthquake damage × After 2017			.42**	.46**		
			(.14)	(.17)		
Distance from earthquake in 100km × After 2017					–.08*	–.07*
					(.03)	(.03)
N	10269	3109	10269	3109	10269	3109
Adj. R^2						
Control variables	No	Yes	No	Yes	No	Yes

Note: Standard errors are in parentheses. Poisson regression with year and municipality fixed effects, and robust standard errors.
*p < 0.05; **p < 0.01; ***p < 0.001. Two-tailed test.

A4.30 Earthquake exposure and homicide

	(1)	(2)	(3)	(4)	(5)	(6)
Within 250km from earthquake × After 2017	–4.67***	–4.19**				
	(1.37)	(1.38)				
Earthquake damage × After 2017			–5.13***	–3.13*		
			(1.37)	(1.30)		
Distance from earthquake in 100km × After 2017					1.07	0.91
					(0.78)	(0.74)
Constant	4.50***	19.48**	4.49***	19.59**	4.49***	19.36**
	(.44)	(6.56)	(.44)	(6.59)	(.45)	(6.42)
N	49434	17225	49434	17225	49434	17225
Adj. R^2	.01	.05	.01	.05	.01	.05
Control variables	No	Yes	No	Yes	No	Yes

Note: Standard errors are in parentheses. Linear regression with year and municipality fixed effects, and municipality clustered standard errors.
*p < 0.05; **p < 0.01; ***p < 0.001. Two-tailed test.

A4.31 Earthquake exposure and robbery

	(1)	(2)	(3)	(4)	(5)	(6)
Within 250km from earthquake × After 2017	52.71*	37.22				
	(24.02)	(27.04)				
Earthquake damage X After 2017			52.70*	54.16*		
			(23.32)	(26.32)		
Distance from earthquake in 100km × After 2017					–19.87**	–19.97***
					(6.25)	(5.80)
Constant	363.97***	477.49***	361.71***	475.56***	363.83***	476.56***
	(23.39)	(110.56)	(23.10)	(111.01)	(23.24)	(110.30)
N	20303	17225	20303	17225	20303	17225
Adj. R^2	.01	.08	.01	.08	.01	.08
Control variables	No	Yes	No	Yes	No	Yes

Note: Standard errors are in parentheses. Linear regression with year and municipality fixed effects, and municipality clustered standard errors.
*p < 0.05; **p < 0.01; ***p < 0.001. Two-tailed test.

A4.32 Distance to active volcano and lynching rate

Different from a one-time shock, long-term exposure to active volcanos can affect the social structure of a community over time, as individuals adapt after experiencing disaster or in anticipation of future disaster (de Mauleón 2023). The advantage of this approach is that I can examine the effect of disaster exposure on community ties in a cross-sectional set-up using the same survey-based measure of community ties as above.

I use distance to volcanos that were active in the past 100 years. Does this variable satisfy the conditions for a plausibly exogenous proxy? First, active volcanos can be seen "as if random" because there is no direct relationship between volcanos and lynching. Second, I can examine whether this proxy is related to community ties, as I have a measure for neighborly cooperation in this cross-sectional dataset (see Models 1, 3, and 5). With increasing distance, there is less neighborly cooperation, as expected. This means distance to volcano predicts community ties. Third, does the proxy influence lynching only through community ties? This question remains open as volcano exposure could have led—through migration, state attention, or soil fertility—to a distinct path for social behavior. This is a central limitation of using volcano exposure for this analysis and needs to be weighed against the collected evidence in the remaining analysis.

The negative coefficients in the below table suggest exposure to active volcanos (less distance) is associated with both more neighborly cooperation, an indicator of community ties, and higher lynching rates. The coefficients of Models 2, 4, and 6 remain in a similar range despite an increasing number of control variables and model fit (R^2).

Given that distance to volcano may not be linearly related to community ties, I also use the squared distance and the square root of distance. Both produce similar results.

	(1) Neighborly cooperation	(2) Lynching rate (log)	(3) Neighborly cooperation	(4) Lynching rate (log)	(5) Neighborly cooperation	(6) Lynching rate (log)
Distance to	–.02***	–.12**	–.01***	–.16***	–.01***	–.13***
active volcano	(.00)	(.04)	(.00)	(.04)	(.00)	(.03)
Trust	.09	–.74	.10*	–.88*	.11*	–.30
	(.04)	(.56)	(.04)	(.41)	(.05)	(.41)
Constant	.36***	1.31*	.33***	3.14**	.37***	2.26
	(.04)	(.48)	(.07)	(.93)	(.10)	(1.17)
N	1589	1837	1589	1837	1557	1704
Adj. R^2	.19	.06	.23	.13	.25	.19
Control variables	No	No	Some	Some	All	All

Note: Standard errors are in parentheses. Linear regression with Estado clustered standard errors.
*$p < 0.05$; **$p < 0.01$; ***$p < 0.001$. Two-tailed test.

A5. References

Agostini, Maximilian, and Martijn van Zomeren. 2021. "Toward a Comprehensive and Potentially Cross-Cultural Model of Why People Engage in Collective Action: A Quantitative Research Synthesis of Four Motivations and Structural Constraints." *Psychological Bulletin* 147(7):667–700.

Aguilar, Claudia. 2019. "Al mes, un linchamiento en Puebla: SGG." *Síntesis Puebla* (blog). April 27 (https://puebla.sintesis.mx/2019/04/27/al-mes-un-linchamiento-puebla/).

Angrist, Joshua D., and Jorn-Steffen Pischke. 2008. *Mostly Harmless Econometrics: An Empiricist's Companion*. Princeton, NJ: Princeton University Press.

Bellingcat. 2018. "How to Prevent, Identify and Address Vicarious Trauma — While Conducting Open Source Investigations in the Middle East." October 18 (https://www.bellingcat.com/resources/how-tos/2018/10/18/prevent-identify-address-vicarious-trauma-conducting-open-source-investigations-middle-east/).

CNDH. 2019. "Informe Especial Sobre Los Linchamientos En México." Mexico: Comisión Nacional de Derechos Humanos.

Davenport, Christian, and Will H. Moore. 2015. "Conflict Consortium Standards & Best Practices for Observational Data." Ann Arbor: University of Michigan.

Gamallo, Leandro. 2015. "Los Linchamientos En México En El Siglo XXI." *Revista Mexicana de Sociología* 77(2):183–213.

García-Sánchez, Miguel, and Rosario Queirolo. 2021. "A Tale of Two Countries: The Functioning of List Experiments to Measure Drug Consumption in Opposite Contexts." *International Journal of Public Opinion Research* 33(2):255–72.

Godínez Pérez, Elisa. 2017. "Linchamientos En México: Entre El Toque de Campana y El Poder Espontáneo." Mexico City: UAM.

Godoy, Angelina Snodgrass. 2006. *Popular Injustice: Violence, Community, and Law in Latin America*. Stanford, CA: Stanford University Press.

Graham, Jesse, Brian A. Nosek, Jonathan Haidt, Ravi Iyer, Spassena Koleva, and Peter H. Ditto. 2011. "Mapping the Moral Domain." *Journal of Personality and Social Psychology* 101(2):366–85.

Huff, Connor, and Dustin Tingley. 2015. "'Who Are These People?' Evaluating the Demographic Characteristics and Political Preferences of MTurk Survey Respondents." *Research & Politics* 2(3).

Kreuter, Frauke, Stanley Presser, and Roger Tourangeau. 2008. "Social Desirability Bias in CATI, IVR, and Web Surveys: The Effects of Mode and Question Sensitivity." *Public Opinion Quarterly* 72(5):847–65.

Mauleón, Héctor de. 2023. "El indomable volcán." *El Universal,* May 24 (https://www.eluniversal.com.mx/opinion/hector-de-mauleon/el-indomable-volcan/).

Mendoza, Carlos. 2008. "Linchamientos en México y Guatemala: reflexiones para su análisis comparado." *El Cotidiano* 152:43–51.

Miller, Erin, Roudabeh Kishi, Clionadh Raleigh, and Caitriona Dowd. 2022. "An Agenda for Addressing Bias in Conflict Data." *Scientific Data* 9(1):593.

Nussio, Enzo, and Govinda Clayton. 2024. "Introducing the Lynching in Latin America (LYLA) Dataset." *Journal of Peace Research*.

Odartey-Wellington, Felix, and Sarah MacRae. 2020. "'Newsworthy Victims': The Killing of Maxwell Mahama and the Culture of Lynching in Ghana." *Journal of Applied Journalism & Media Studies* 9(3):307–26.

Rodríguez Guillén, Raúl, and Norma Ilse Veloz Ávila. 2019. "Linchamientos En México: Una Puesta al Día." *El Cotidiano* 34(214):87–94.

Skinner, Chris J. 2016. "Probability Proportional to Size (PPS) Sampling." Pp. 1–5 in *Wiley StatsRef*. New York: John Wiley & Sons, Ltd.

Spilerman, Seymour, and Emanuele Gerratana. 2009. "The Time Series of Lynchings in the American South." Pp. 176–90 in *A Quantitative Tour of the Social Sciences*, edited by A. Gelman and J. Cortina. Cambridge, UK: Cambridge University Press.

Sundberg, Ralph, and Erik Melander. 2013. "Introducing the UCDP Georeferenced Event Dataset." *Journal of Peace Research* 50(4):523–32.

UNODC. 2010. "Manual on Victimization Surveys." Geneva: UNODC (https://www.unodc.org/documents/data-and-analysis/Crime-statistics/Manual_on_Victimization_surveys_2009_web.pdf).

Vilalta, Carlos J., Robert Muggah, and Gustavo Fondevila. 2020. "Homicide as a Function of City Block Layout: Mexico City as Case Study." *Global Crime* 21(2):111–29.

Weidmann, Nils B. 2015. "On the Accuracy of Media-Based Conflict Event Data." *Journal of Conflict Resolution* 59(6):1129–49.

Wilkinson, Mark D., Michel Dumontier, IJsbrand Jan Aalbersberg, Gabrielle Appleton, Myles Axton, Arie Baak, Niklas Blomberg, et al. 2016. "The FAIR Guiding Principles for Scientific Data Management and Stewardship." *Scientific Data* 3(1):160018.
